# Transcriptional Slippage and RNA Editing Increase the Diversity of Transcripts in Chloroplasts: Insight from Deep Sequencing of *Vigna radiata* Genome and Transcriptome

**DOI:** 10.1371/journal.pone.0129396

**Published:** 2015-06-15

**Authors:** Ching-Ping Lin, Chia-Yun Ko, Ching-I Kuo, Mao-Sen Liu, Roland Schafleitner, Long-Fang Oliver Chen

**Affiliations:** 1 Institute of Plant and Microbial Biology, Academia Sinica, Nankang, Taipei 11529, Taiwan; 2 Department of Earth and Life Science, University of Taipei, Taipei 10048, Taiwan; 3 AVRDC—The World Vegetable Center, Shanhua, Tainan 74199, Taiwan; NIH, UNITED STATES

## Abstract

We performed deep sequencing of the nuclear and organellar genomes of three mungbean genotypes: *Vigna radiata ssp*. *sublobata* TC1966, *V*. *radiata* var. *radiata* NM92 and the recombinant inbred line RIL59 derived from a cross between TC1966 and NM92. Moreover, we performed deep sequencing of the RIL59 transcriptome to investigate transcript variability. The mungbean chloroplast genome has a quadripartite structure including a pair of inverted repeats separated by two single copy regions. A total of 213 simple sequence repeats were identified in the chloroplast genomes of NM92 and RIL59; 78 single nucleotide variants and nine indels were discovered in comparing the chloroplast genomes of TC1966 and NM92. Analysis of the mungbean chloroplast transcriptome revealed mRNAs that were affected by transcriptional slippage and RNA editing. Transcriptional slippage frequency was positively correlated with the length of simple sequence repeats of the mungbean chloroplast genome (R^2^=0.9911). In total, 41 C-to-U editing sites were found in 23 chloroplast genes and in one intergenic spacer. No editing site that swapped U to C was found. A combination of bioinformatics and experimental methods revealed that the plastid-encoded RNA polymerase-transcribed genes *psbF* and *ndhA* are affected by transcriptional slippage in mungbean and in main lineages of land plants, including three dicots (*Glycine max*, *Brassica rapa*, and *Nicotiana tabacum*), two monocots (*Oryza sativa* and *Zea mays*), two gymnosperms (*Pinus taeda* and *Ginkgo biloba*) and one moss (*Physcomitrella patens*). Transcript analysis of the *rps2* gene showed that transcriptional slippage could affect transcripts at single sequence repeat regions with poly-A runs. It showed that transcriptional slippage together with incomplete RNA editing may cause sequence diversity of transcripts in chloroplasts of land plants.

## Introduction

The chloroplast (CP) genome originated from the genome of endosymbiontic cyanobacteria-like photosynthetic bacteria [1–4]. Most genes of the primitive CPs were transferred to the plant nuclear genome [5–7], consequently the modern CP genome is highly reduced in both size and gene content. Only about 4 rRNAs, 30 tRNAs and 100 protein-coding genes are retained in 120~210-kb DNA of CP genomes [8,9]. The CP genes carry out functions in photosynthesis (e.g., *atp*, *pet*, *ndh*, *psa* and *psb*), or are involved with bacteria-like transcription (e.g., *rpoA*, *B*, *C1*, *C2*) and translation (e.g., *rpl*, *rps*, *tRNAs*, *rRNAs*).

In bacteria such as *Escherichia coli*, transcriptional slippage (TS) occurs at simple sequence repeat (SSR) regions, especially at poly-A or poly-T runs [10,11]. TS is mediated by the bacterial-type RNA polymerase, which leads to the incorporation of more or less nucleotides into the transcript, thereby producing a population of heterogeneous mRNA sequences. The heterogeneous transcripts include RNA sequences that are inconsistent with their DNA templates. If TS occurs in open reading frames, RNA transcripts of a gene are expected to encode different protein products. Both plastid-encoded RNA polymerase (PEP) and nuclear-encoded RNA polymerases (NEP) are active in CPs [12]. Unlike the phage-type NEP, PEP has a core enzyme like *E*. *coli* RNA polymerase [13,14], so TS is likely to occur in CPs. TS has been reported to occur in endosymbiontic bacteria such as *Buchnera aphidicola* and *Blochmannia pennsylvanicus* [15], but has not yet been found in CPs. Recently, several RNA-seq-based papers showed the genome-wide view of transcript variability in *Arabidopsis thaliana* [16–18] and ferns [19], but these studies focused on only RNA editing (RE), not TS.

RE is a post-transcriptional modification in CPs that can change C to U residues or vice versa at specific sites of RNAs in both coding and noncoding regions, producing transcripts that are inconsistent with their DNA templates [20,21]. RE in coding regions can alter the amino acid sequence of proteins. For example, editing of the second position of the ACG codon at 5’ end of transcripts will create an AUG initiation codon [22,23], and editing of the first position of CAA, CAG and CGA will create stop codons [23]. RE is common in CPs and editing patterns have been studied in crops such as maize [24,25], sugarcane [26], rice [27], pea [28], tomato [26], cotton [29] and black pine [30].

Mungbean (*Vigna radiata* L.), an increasingly important legume crop, is currently grown on 6 million hectares, mainly in Asia, but also in Australia and Canada. Genomics studies of this crop lag behind that of many other plants, although because of its short generation time and relative small genome size, mungbean could be a model legume plant for genomic analyses. In the present study, we used mungbean as a model plant to investigate TS and RE in CP genes. We compared the CP genomes of three mungbean genotypes based on the deep sequencing results and analysed low frequency sequence variations in the transcriptome of mungbean RIL59. RT-PCR, cloning and Sanger sequencing were used to validate the findings. The results were compared to CP transcriptome variations in the major lineages of land plants.

## Materials and Methods

### Plant material and genomic DNA purification

The seeds of *Vigna radiata* var. *sublobata* TC1966, *V*. *radiata* var. *radiata* NM92 and RIL59, an F_11_ recombinant inbred line derived from a cross of TC1966 x NM92, were obtained from AVRDC—The World Vegetable Center, Taiwan. The seed was germinated and planted in the greenhouse of the Institute of Plant Molecular Biology, Academia Sinica, Taiwan. Genomic DNA (gDNA) of TC1966, NM92 and RIL59 was extracted from 0.5 g of young leaves harvested from seedlings with use of the Plant Genomic DNA Extraction Minprep kit (Viogene-Biotek Corp. New Taipei City, Taiwan) following the manufacturer’s instructions. The integrity and purity of the extracted gDNAs were confirmed by gel electrophoresis and spectroscopy verifying that the ratio 260 to 280 nm is > 1.8 and the 260 to 230 nm is > 2.0.

### Whole genome sequencing and read assembly

The qualified gDNAs of mungbean lines were sequenced on an Illumina HiSeq 2000 sequencer (Genomics BioSci & Tech Co., New Taipei City, Taiwan). Paired-end reads (2 x 100 bp) trimmed with an error probability < 0.05 were collected for genome assembly. The CP genome was assembled by use of MIRA 4 [31] and MITObim 1.7 [32]. *V*. *radiata* var. *radiata* KPS1 CP genome (GenBank accession no. GQ893027 [33]) was used as a reference. The workflow of MITObim was described in detail in [33]. The CP draft sequences generated from MITObim were re-mapped by using bowtie2 2.2.1 [34] to check the accuracy of assembly. Re-mapping alignments were visualized with use of Integrative Genomics Viewer 2.3.32 [35,36], then manually inspected. Sites and regions with a depth lower than 1/100 of the average coverage or ambiguous sequences were considered as gaps and were verified by PCR and Sanger sequencing. The boundaries of inverted repeats (IRs) and single copy regions (SCs) were verified by PCR and Sanger sequencing. Calculation of the genome coverage and conversion of alignment formats were carried out by using SAMtools 1.1 [37]. The CP sequences were deposited at DDBJ (DNA Data Bank of Japan).

### Sequence annotation

Protein coding, ribosomal RNA (rRNA), and tRNA genes were annotated by use of DOGMA [38] and the annotation was confirmed by BLAST searches (http://blast.ncbi.nlm.nih.gov/Blast.cgi). All tRNA genes were further verified by use of tRNAscan-SE 1.21 [39]. The genome map was drawn with use of Circos 0.67 [40]. CP SSRs with length more than 8 bp were identified by use of MISA [41] with default parameters. The CP genomes of different mungbean lines were aligned by use of bl2seq included in NCBI BLAST (http://blast.ncbi.nlm.nih.gov/Blast.cgi).

### RNA extraction and RNA-seq

Total RNA was extracted from a whole plant of 1-month-old RIL59 seedling with a modified CTAB-based method [42]. The gDNA contaminants were removed from extracted RNA by use of Ambion TURBO DNA-free Kit (Life Technologies Co.). DNA-removed RNA samples were analysed on an RNA LabChip with Agilent 2100 Bioanalyzer. Samples with >7.0 RNA integrity, >1.4 rRNA ratio, and no DNA contamination were selected for experiments. The qualified RNA underwent ribosomal RNA depletion with TruSeq Stranded Total RNA with Ribo-Zero Plant Kit (Illumina, San Diego, CA, USA). The cDNA libraries were generated by RNA fragmentation, random hexamer-primed cDNA synthesis, linker ligation and PCR amplification. RNA-seq was carried out on an Illumina HiSeq 2000 system (Genomics BioSci & Tech Co., New Taipei City, Taiwan). Three criteria were used to filter dirty raw reads: remove reads with sequence adaptors; remove reads with > 2% 'N' bases; and remove low-quality reads, which have more than half of bases with QA ≤ 15. The RNA-seq data of RIL59 was uploaded to the NCBI Sequence Read Archive (accession no. SRR1867748).

### Identification of transcript diversity

The clean reads were mapped to the sequence of the RIL59 CP genome with use of TopHat2 [43]/bowtie2 [34]. The parameters for TopHat2 used in mapping were set to “-N 2—read-gap-length 2—max-insertion-length 3—max-deletion-length 3”. The RNA-seq alignment was visualized by use of Integrative Genomics Viewer 2.3.32 [35,36], then manually inspected. The RE was inferred from C-to-U substitutions with a cut-off value of at least 5% edited reads. Indel-containing reads in every SSR locus were assumed to be results from TS. The editing ratio and TS frequency were inferred from the number of substitution and indel reads to total aligned reads at RE and TS sites, respectively.

### Verification of TS and RE sites

First strand cDNA was synthesized with use of SuperScript III Reverse Transcriptase (Invitrogen) following the manufacturer's protocol with gene-specific primers (S1 Table). PCR and RT-PCR were performed with use of a high fidelity Taq polymerase *Pfu* (Invitrogen). Amplicons of RT-PCR were directly sequenced by use of ABI PRISM 3730xl. To further verify the TS and RE ratio, RT-PCR amplicons were cloned, then sequenced using ABI PRISM 3730xl. The TS and RE ratio was inferred from the ratio of different sequences of clones.

## Results and Discussion

### Sequencing and general features of the mungbean CP genome

**Fig 1:**
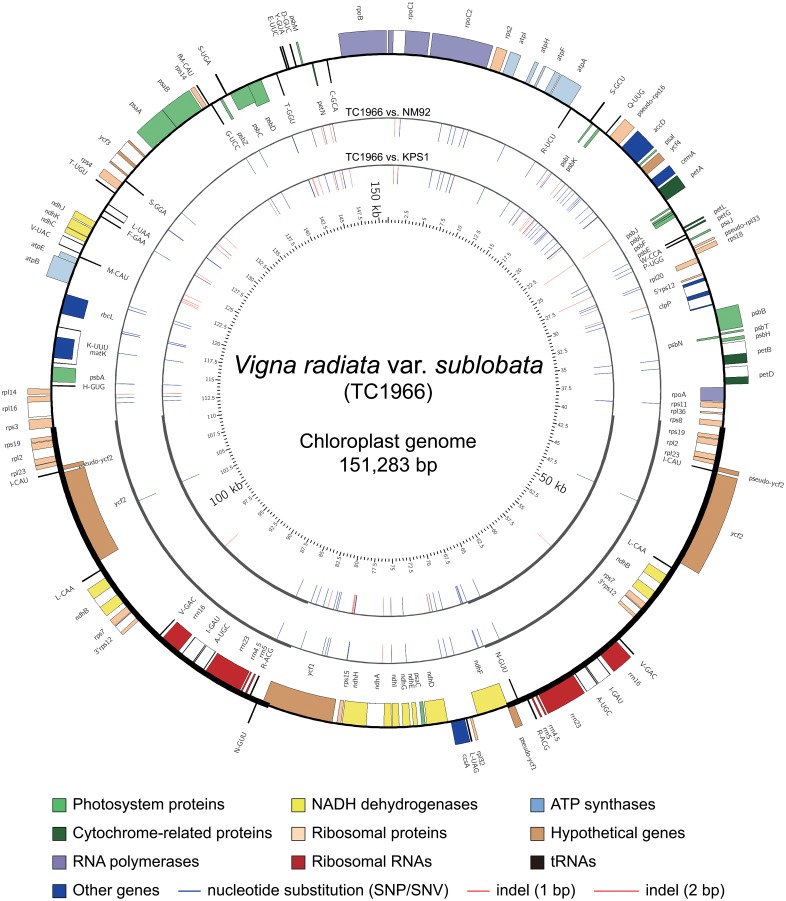
CP genome map and comparisons of *Vigna radiata* var. *sublobata* TC1966. Four concentric circles from the outside to inside represent the TC1966 CP genome map, the variances between TC1966 and NM92 (RIL59), the variances between TC1966 and KPS1, and the length scale, respectively. The histograms on the outer and the inner circles of the TC1966 CP genome map denote the clockwise and the anticlockwise transcription of the genes, respectively.

The whole genome shotgun sequencing (WGS) of three mungbean lines, NM92, TC1966 and RIL59, generated 34.17, 37.96 and 154.48 million clean paired-end reads, respectively. Through baiting and iterative mapping, CP sequence reads were extracted from the total read pools and assembled into whole CP genomes. The achieved read depth of the CP genomes of TC1966, NM92, and RIL59 were on average 5,168-, 6,409- and 7,914-fold (S2 Table), respectively. We compared the CP genomes of wild mungbean TC1966 (DDBJ accession no. AP014692), mungbean line NM92 (DDBJ accession no. AP014691), RIL59 (this study) and line KPS1 [33]. The length of the TC1966 CP genome was 151,283 bp, which is 12 bp longer than the CP genome of NM92 and RIL59 (Fig 1). Both NM92 and RIL59 CP genomes have identical sequences, of 151,281 bp, and are 10 bp larger than the CP genome sequence of KPS1. In total, 213 SSRs, including one dinucleotide repeat and 212 single nucleotide repeats, were identified in the CP genomes (S3 Table). Overall, 108 of the single nucleotide repeats are 8-mers, 66 are 9-mers, 17 are 10-mers, 12 are 11-mers, and 5 are 12-mers. SSRs from 13-mer to 16-mer represent one single nucleotide repeat each.

The mungbean CP genome has a quadripartite structure (Fig 1): a pair of inverted repeats (IRs) is separated by a large single copy (LSC) and a small single copy (SSC). The sizes of the IRs, LSC and SSC in the CP genomes of NM92 (RIL59), TC1966 and KPS1 differ. In the cultivated mungbean NM92 and RIL59, the IR, LSC and SSC contain 26,475, 80,901 and 17,430 bp, respectively, whereas in TC1966, these elements are 26,482 bp, 80,904 bp and 17,415 bp, respectively. CP genomes of TC1966, NM92 and RIL59 are predicted to encode 126 genes that are consistent with those in KPS1 [33]. Eighteen of these genes are duplicated in IRs: 7 protein-coding genes (*rps19*, *rpl2*, *rpl23*, *ycf2*, *ndhB*, *rps7* and 3’*rps12*), 7 tRNA genes (*I*-*CAU*, *L*-*CAA*, *V*-*GAC*, *I*-*GAU*, *A*-*UGC*, *R*-*ACG* and *N*-*GUU*), and 4 rRNA genes (*rrn16*, *rrn23*, *rrn4*.*5* and *rrn5*). Overall, 107 unique genes, including 29 tRNAs, 4 rRNAs and 74 proteins, are present as single copies in the mungbean CP genome.

### SNVs, indels, and possibility of paternal leakage

The four mungbean CP genomes were aligned for detailed sequence comparison. Between TC1966 and NM92 (RIL59) CP genomes, 78 single nucleotide variations (SNVs) and 9 indels contribute a 78-bp and 10-bp difference (S4 and S5 Tables), respectively. In total, 88 bp distinguish the CP genomes of TC1966 from NM92 (RIL59). Of the 78 SNVs, 13 are transitions and 65 are transversions. Of the nine indels, eight are 1-bp and one is 2-bp. As expected, the CP genome of the cultivated variety KPS1 is more distant from wild mungbean TC1966 than the other two cultivated mungbean genotypes NM92 and RIL59 (S4 and S5 Tables). The KPS1 CP genome contains all variants that distinguish NM92 (RIL59) from TC1966 and has 32 additional 1-bp indels as compared with TC1966 (Fig 1). Uniparental transmission of the CP genome via the female gametophyte is the most common mode of inheritance in angiosperms [44], but a low-frequency leakage of plastids via pollen seems to be universal in plants. Paternal leakage was assessed for the cross NM92 x TC1966 by in-depth comparison of the CP genome sequence polymorphisms between the parents and RIL59. The RIL59 CP genome was identical to its maternal parent NM92. No heteroplasmy pattern was observed in RIL59 after assessing all SNVs and indels documented for comparing of the CP genomes of TC1966 and NM92 at a sequencing depth from 7,762- to 7,998-fold. The absence of TC1966-specific variants suggested that the possibility of paternal leakage is low or even absent in RIL59.

### Transcriptional slippage in the mungbean CP genome is positively correlated with SSR length

**Fig 2:**
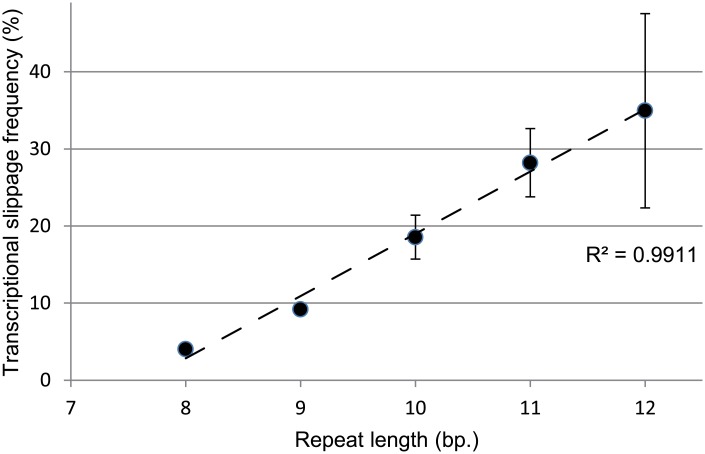
Correlation between transcriptional slippage (TS) frequency and simple sequence repeat (SSR) length.

TS takes place in bacteria at SSR tracts and alters the transcript size at SSRs. To study the TS pattern in the mungbean CP genome, we assessed transcript diversity at all SSRs present in the CP genome. For 213 CP SSRs, variation in SSR sequence length was observed in 0 to 73.86% of all transcripts from SSR-containing loci (S6 Table). The 13-, 14-, 15- and 16-bp-long SSRs presented in single locus. Excluding the single-locus SSRs that lack replicates in statistical analyses, the mean TS frequency was positively correlated with the length of the corresponding SSR motif (R^2^ = 0.9911, Fig 2). Although the R^2^ is close to 1, the standard errors are relative higher for longer SSRs, indicating TS frequency tends to be highly variable in longer SSRs. This finding suggested that several polymerases with different fidelity are subject to errors at longer SSR transcripts. The polymerases involved in this phenomenon are suggested to be PEP, NEP, and even the polymerases used in the RNA-seq experiments [45,46]. This suggestion is reasonable because genes in CP are transcribed by PEP, NEP only or both [12].

Moreover, Taq polymerases and reverse transcriptases are possibly subject to errors at SSRs [45,46]. In order to minimize the risk that the indels detected in the transcriptome sequences were experimental artefacts, high fidelity polymerases such as *Pfu* and SuperScript III Reverse Transcriptase were used in the verification experiments. Nevertheless it cannot be excluded that indels introduced by the polymerase and reverse transcriptase contributed to the observed transcript variability.

### Identification of RE sites

RE sites were identified as mismatches between RNA-seq reads and the CP genome sequence. In total, 41 C-to-U editing sites were detected in transcripts of 23 CP genes (Table 1), with no U-to-C editing identified. The editing sites were almost exclusively located in protein-coding regions, only one site was found in the intergenic region between *rpl20* and 5’*rps12* genes. Five editing sites altered the first nucleotide of a codon, 34 sites the second, and one site the third nucleotide of a codon. Altogether, 39 nonsynonymous and one synonymous substitutions were generated in the RIL59 CP genome by RE. The editing level was inferred from the C versus U ratio of the transcripts derived from the respective loci (Table 1). Three sites located in *atpA*, *ndhD* and the intergenic region between *rpl20* and 5’*rps12* were edited at the lowest observed frequency (< 33.3%). Five sites located in *psbN*, *rpoA*, *petL*, *rpoB* and *rpoC1* showed moderate editing levels (33.3–66.7%) and the remaining sites were edited at high levels (> 66.7%).

**Table 1:** RNA editing pattern in the CP genome of mungbean accession RIL59.

Gene	Genome		Codon		Codon position	Amino acid		Editing level
	Position	Strand	from	to		from	to	U/C (%)
*ndhC*	13922	+	UCA	UUA	2	S	L	84
*rps14*	26562	+	UCA	UUA	2	S	L	97
*rpoB*	37461	+	UCU	UUU	2	S	F	59
*rpoB*	37674	+	UCA	UUA	2	S	L	68
*rpoB*	37689	+	UCG	UUG	2	S	L	80
*rpoB*	39123	+	UCU	UUU	2	S	F	73
*rpoC1*	40403	+	UCA	UUA	2	S	L	84
*rpoC1*	41679	+	UCA	UUA	2	S	L	67
*rps2*	47981	+	ACA	AUA	2	T	I	97
*rps2*	48095	+	UCA	UUA	2	S	L	97
*atpF*	51307	+	CCA	CUA	2	P	L	98
*atpA* ^1^	53350	+	UCC	UCU	3	S	S	6
*accD*	59680	+	UCG	UUG	2	S	L	96
*psaI*	60699	+	CAU	UAU	1	H	Y	91
*psbF*	64845	-	UCU	UUU	2	S	F	96
*psbE*	64969	-	CCU	UCU	1	P	S	98
*petL*	66108	+	CCC	CUC	2	P	L	57
*rps18*	68155	+	UCG	UUG	2	S	L	82
Spacer*	68964	+	C	U	-	-	-	27
*clpP*	70022	-	CAU	UAU	1	H	Y	90
*psbN*	74413	-	UCU	UUU	2	S	F	31
*petB*	76357	+	UCA	UUA	2	S	L	98
*rpoA*	78836	-	UCA	UUA	2	S	L	35
*rpl23*	83522	-	UCA	UUA	2	S	L	74
*ndhB*	92403	-	CCA	CUA	2	P	L	89
*ndhB*	92629	-	CAU	UAU	1	H	Y	95
*ndhB*	92772	-	UCA	UUA	2	S	L	93
*ndhB*	93048	-	UCA	UUA	2	S	L	84
*ndhB*	93054	-	UCA	UUA	2	S	L	68
*ndhB*	93830	-	UCU	UUU	2	S	F	92
*ndhB*	93839	-	CCA	CUA	2	P	L	84
*ndhB*	93990	-	CAU	UAU	1	H	Y	87
*ndhB*	94034	-	ACG	AUG	2	T	M	83
*ndhB*	94427	-	UCA	UUA	2	S	L	96
*ndhD*	112418	-	UCA	UUA	2	S	L	77
*ndhD*	112622	-	UCA	UUA	2	S	L	95
*ndhD*	112913	-	ACA	AUA	2	T	I	97
*ndhD*	113294	-	ACG	AUG	2	T	M	14
*ndhE*	114034	-	CCG	CUG	2	P	L	86
*ndhA*	115872	-	UCU	UUU	2	S	F	84
*ndhA*	117875	-	UCA	UUA	2	S	L	93

* denotes a synonymous substitution.

### U-indels overlapping with RE sites appear in transcripts of *psbF*

**Fig 3:**
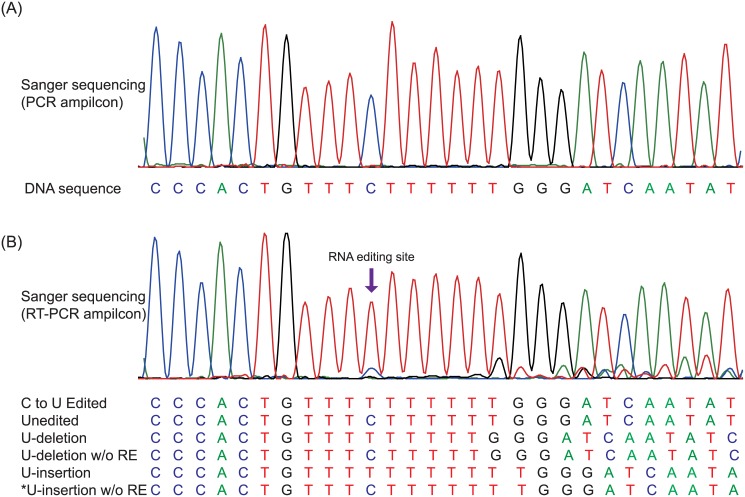
RNA transcript variation of *psbF* gene. (A) CP genome sequence detected by direct sequencing of PCR amplicons. (B) The sequencing “noise” at the U-indel and RNA editing (RE) sites of the transcripts detected by direct sequencing of RT-PCR fragments. Five *psbF* sequence variants were obtained after sequence analysis of cloned RT-PCR amplicons. One predicted sequence, which could not be detected by sequencing of clones, is marked with a star.

RNA-seq data suggested that 12.03% of *psbF* transcripts had a U-insertion or U-deletion near the editing site (S1 Fig). The observation was verified by RT-PCR and Sanger sequencing. The RT-PCR products showed sequencing “noise” at the editing sites and the end of the SSR (Fig 3). For further confirmation of the U-indels, the amplicons were cloned and 62 independent colonies were randomly chosen for Sanger sequencing. From these clones, 47 (75.81%) showed C to U editing, 5 (8.06%) had unedited sequences, and U-insertion and U-deletion sequences were found in 4 (6.45%) and 5 (8.06%) clones, respectively. One of the clones (1.61%) had an unedited C at the RE position but a U deletion nearby (Fig 3 and S2 Fig). The similar percentage of indel revealed in the transcripts in both RNA-seq data and experimental verification with use of high fidelity polymerases suggests that TS indeed subjects indels at the locus rather than an artefact from Taq polymerase.

### Origin of the U-indel containing transcripts

Horizontal gene transfer from CPs to the nucleus or to mitochondria has been documented for plants [47]. Nuclear plastid like (NUPT) and mitochondrial plastid like (MTPT) sequences generally accumulated substitutions and indels during their evolution. To test whether the U-indel *psbF* transcripts were derived from NUPTs or MTPTs and therefore not from the CP genome, we used bowtie2 with default parameters for mapping WGS and RNA-seq reads to the CP genome. The WGS reads, comprising 15.45 Gbp, covered the 579 Mbp mungbean genome [48] approximately 26.7-fold. For WGS read mapping, if reads from NUPTs and MTPTs were mapped to the CP genome, their depths should be approximately equal to or many times greater than 26.7-fold, depending on the copy numbers of NUPTs and MTPTs in mungbean. All WGS reads corresponding to *psbF* mapped to the CP genome without any U-indel (S3 Fig). In contrast, RNA-seq reads mapped by the same procedure showed C-to-U substitution and U-indel patterns (S3 Fig), which strongly indicates that the U-indels are derived from transcriptional processes at the CP genome level, not from NUPTs or MTPTs, which agrees with the *psbF* variants derived from the CP genome.

### Relation of U-indels in *psbF* transcripts to RE

**Fig 4:**
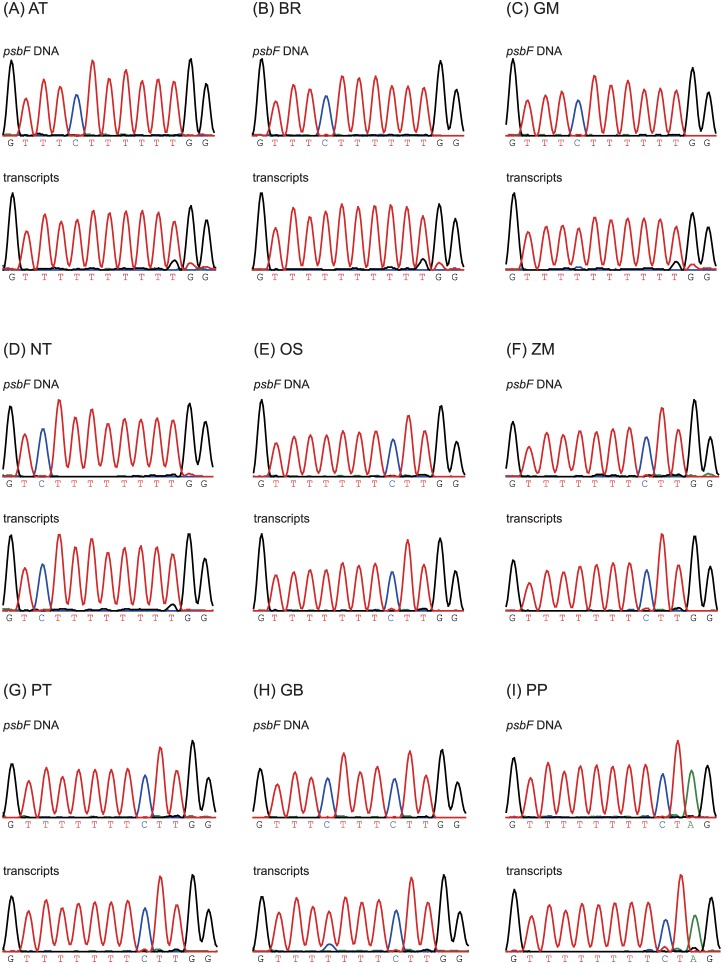
*PsbF* CP genome sequence and *psbF* transcript variation in main lineages of land plants. Sequence chromatograms of PCR and RT-PCR amplicons of *psbF* from *Arabidopsis thaliana* (AT), *Brassica rapa* (BR), *Glycine max* (GM), *Nicotiana tabacum* (NT), *Oryza sativa* (OS), *Zea mays* (ZM), *Pinus taiwanensis* (PT), *Ginkgo biloba* (GB), and *Physcomitrella patens* (PP) showing the RE and TS sites (as sequencing “noise”) in mapped transcripts.

C-to-U and U-to-C substitutions by RE are known processes in plant organelles [24,25], whereas U-indels have been detected in mitochondrial transcripts of Trypanosomatids and Myxomycota [49]. U-indels have not yet been analyzed in-depth for plants. To test whether U-indels in *psbF* transcripts are related to RE, we searched for evidence for these features in publicly available RNA-seq data for land plants. Transcriptome sequence data for four dicot species (Rosid: *Glycine max*, *Arabidopsis thaliana*, *Brassica rapa*; and Asterid: *Nicotiana tabacum*), two monocot species (*Oryza sativa* and *Zea mays*), two gymnosperm species (*Pinus taeda* and *Ginkgo biloba*) and a moss (*Physcomitrella patens*) available from the Sequence Read Archive (SRA) at NCBI were analyzed for presence of U-indels and RE in *psbF* transcripts (S7 Table). All tested Rosid species and *G*. *biloba* but not the remaining species shared the same editing site in *psbF* [50]. The SRA data for these nine species were mapped to the *psbF* sequences of the corresponding CP genomes, and the C-to-U editing pattern in these nine species was consistent with previous studies [50] (S4 Fig). Interestingly, the U-insertion and U-deletion patterns were present in tested species independent of whether the C-to-U RE site was present or not (S4 Fig). The U-insertion and U-deletion patterns in *G*. *biloba* and *P*. *patens* are possibly due to insufficient quantity of RNA-seq reads because the SRA datasets were generated from poly-A enrichment methods, not from rRNA depletion methods. The RNA-seq mapping results were validated by Sanger sequencing, which resulted in a similar sequence “noise” in *psbF* transcripts for *G*. *max*, *A*. *thaliana*, *B*. *rapa* and *N*. *tabacum* as in mungbean (Fig 4) and a weak “noise” peak in tested monocots, gymnosperms and moss.

To further verify this finding, we randomly cloned and sequenced 80 of *psbF* RT-PCR amplicons from *N*. *tabacum*, which lacks the RE site in *psbF*, and found that two clones had an U-insertion and one clone an U-deletion (S5 Fig). Additionally, the *psbF* amplicons derived from *P*. *patens*, the most distant species of the chosen land plant set, were selected for cloning to ensure U-indels. From 155 sequenced *psbF* clones for this species, two clones (1.3%) showed U-indels at the poly-U SSR of the gene (S6 Fig). From the presence of U-indels in *psbF* transcripts for the two species that lack the RE site in this gene we concluded that C-to-U RE and U-indel generation in this gene are independent of each other. The sequence surrounding the RE site in *psbF* contains nine continual T residues in mungbean, suggesting that U-indels in *psbF* transcripts are generated by TS.

### Sequence variation in *psbF* transcripts might lead to at least five different proteins sharing the same N-terminal

The *psbF* gene is part of the *psbE* operon, which contains *psbE*, *psbF*, *psbL* and *psbJ* genes. The *psbF* transcripts encode a 39 amino acid protein, with an edited phenylalanine or an unedited serine codon in the 77th nucleotide. U-indels in *psbF* transcripts lead to frame shifts, resulting in aberrant termination of translation. U-insertion at this site in *psbF* transcripts would result in a longer protein variant with 42 instead of 39 amino acid residues. The first 27 N-terminal amino acid residues of this variant would correspond to those of the common *psbF* protein, whereas the 12 following amino acid residues would be altered by the frame shift. The common *psbF* stop codon would be bypassed, thus resulting in a *psbF* variant with three additional amino acid residues. U-deletion transcripts would also bypass the common *psbF* stop codon and extend the *psbF* open reading frame to the stop codon of *psbL*, thus resulting in a *psbF*-*psbL* fusion protein with 85 amino acids in length. Because RE and TS are independent mechanisms, six different *psbF* transcripts are expected to be generated, but only five were identified by our experiments (Fig 3). Further research at the protein level is needed to test whether every different transcript can be translated. Also, if transcripts are translated, the functions of the *psbF* protein variants *in vivo* would be of interest.

### TS patterns in *ndhA* and *rps2* genes

The *psbE* operon is conserved in land plants [51] and is mainly transcribed by PEP, an *E*. *coli*-like RNA polymerase. Similar to the *psbF* transcripts, TS may occur in other PEP transcribed genes. Like *psbF*, the *ndhA* is also mainly transcribed by PEP. The *ndhA* gene in land plants is conserved, except Pinaceae lack all *ndh* genes [52]. In addition, the *ndhA* has high TS frequency (22.37%) at a poly-U SSR with 9 bp in size (S6 Table). Therefore, we assessed the TS patterns of the *ndhA* gene in the main lineages of land plants using both bioinformatic and experimental methods.

Although the SSR in *ndhA* is relatively short, 2.24% reads still revealed indels at WGS mapping result (S7 Fig). We searched the *ndhA* sequence against whole nuclear [53] and mitochondrial genomes [54] of mungbean by using blastn. Results showed that *ndhA*-like sequences were not found in mungbean nuclear and mitochondrial genomes. This suggested that the indel-reads originate from CP genome and possibly generated from a non-proofreading Taq used in library construction during WGS. A clear Sanger sequencing signal of amplicon from a proofreading *Pfu* further supported this suggestion (S7 Fig).

The RNA-seq mapping results show an apparent signal of TS in *V*. *radiata* and *G*. *max*, but no or little signal in *A*. *thaliana*, *B*. *rapa*, *N*. *tabacum*, *O*. *sativa*, *Z*. *mays*, *G*. *biloba* and *P*. *patens* (S8 Fig). The reasons for lacking TS signals may be that the SSR was interrupted by a nucleotide in *A*. *thaliana*, *B*. *rapa*, *N*. *tabacum*, *O*. *sativa*, *Z*. *mays* and *G*. *biloba*, and the insufficient reads was aligned in *P*. *patens*. Sanger sequencing of RT-PCR amplicons was performed to verify this suggestion (S9 Fig). The “noise” peaks of sequencing were revealed in *V*. *radiata*, *G*. *max* and *P*. *patens* but not in *A*. *thaliana*, *B*. *rapa*, *N*. *tabacum*, *O*. *sativa*, *Z*. *mays* and *G*. *biloba*.

Interestingly, a poly-A SSR in the *ndhA* intron of *A*. *thaliana* had a 6.3% of A-indel reads at that SSR. The *rps2* gene was selected to ensure whether TS could occur at poly-A type SSRs. The SSR region in *rps2* gene is conserved in Fabaceae but not in other land plants. Similar to the *ndhA* gene, 4.78% reads showed indels from mungbean WGS mapping result of *rps2* gene (S10 Fig). Also, the results from blastn and amplicon sequencing supported that the *rps2* gene only present in CP genome and the indel-read possibly generated from a non-proofreading Taq.

At the results of RT-PCR amplicon sequencing, as expected, the “noise” peaks are revealed only in Fabaceae species, *V*. *radiata* and *G*. *max* (S11 Fig). Altogether, TS is associated with the SSR, especially in genes transcribed by PEP. Therefore, indels at SSR sites caused by TS are a common feature in land plants.

## Conclusions

The study demonstrated that deep RNA-seq is a powerful tool to obtain a detailed view of transcript variability and can detect sequence variations caused by TS and RE. TS is a common phenomenon in CP transcripts of plants. In mungbean, TS frequency in CP transcripts is highly positively associated with the CP SSR length. TS in CP transcription resembles TS in bacteria [10,11] and TS in CP may be a relict from the bacteria origin of these organelles. Our results demonstrated that TS and RE caused transcript diversity in the CP genome. The implication of the diversity on translation and protein function remains to be elucidated.

## Supporting Information

S1 Fig Alignments of RNA-seq reads at the *psbF* gene of mungbean accession RIL59 by using TopHat2. The editing site is flanked by two vertical black lines. The read sequence at the flanked site is calculated and shown in the black square. The horizontal black line indicates deletions in a read, and the purple spots show insertions in overlapping reads.
(PDF)

S2 Fig Sequence chromatograms of cloned mungbean *psbF* transcripts. Five kinds of sequences were identified: normal transcripts without (A) or with (B) editing, U-deletion transcripts without (C) or with (D) editing, and U-insertion transcripts with editing (E).
(PDF)

S3 Fig Alignments of WGS and RNA-seq reads at the *psbF* gene of mungbean accession RIL59 by using bowtie2. WGS (A) and RNA-seq (B) reads were mapped to the *psbF* gene. The editing site is flanked by two vertical dashed lines. The read sequence at the flanked site is calculated and shown in the black square. The horizontal black line indicates deletions in a read, and the purple spots show insertions in overlapping reads.
(PDF)

S4 Fig Alignment of RNA-seq reads at the *psbF* gene. *G*. *max* (A), *A*. *thaliana* (B), *B*. *rapa* (C), *N*. *tabacum* (D), *O*. *sativa* (E), *Z*. *mays* (F), *P*. *taeda* (G), *G*. *biloba* (H), and *P*. *patens* (I) were included in the analysis. The horizontal black line indicates a deletion, and the purple spot indicates an insertion. The black square denotes the count of read sequences at the site flanked by two vertical dashed lines.
(PDF)

S5 Fig Sequence chromatograms of cloned tobacco *psbF* transcripts. Four kinds of sequences were identified: normal transcripts without (A) or with (B) editing, U-deletion transcripts without (C) editing, and U-insertion transcripts without editing (D).
(PDF)

S6 Fig Sequence chromatograms of cloned *Physcomitrella psbF* transcripts. Three kinds of sequences were identified: normal transcripts (A), U-deletion transcripts (B) and U-insertion transcripts (C).
(PDF)

S7 Fig WGS, RNA-seq mapping and PCR amplicon sequencing at *ndhA* gene. The alignments of WGS (A) and RNA-seq (B) were carried out by bowtie 2 and TopHat2, respectively. PCR amplicon (C) generated from high fidelity *Pfu* was directly sequenced using ABI PRISM 3730xl.
(PDF)

S8 Fig Alignments of RNA-seq reads at the *ndhA* gene by using bowtie 2. *V*. *radiata* (A), *G*. *max* (B), *A*. *thaliana* (C), *B*. *rapa* (D), *N*. *tabacum* (E), *O*. *sativa* (F), *Z*. *mays* (G), *G*. *biloba* (H), and *P*. *patens* (I) were included in the analysis. The black line indicates a deletion, and the purple I character indicates an insertion. The black square denotes the count of read sequences at the site flanked by two vertical dashed lines.
(PDF)

S9 Fig Sequence chromatograms of *ndhA* RT-PCR amplicons. *V*. *radiata* (A), *G*. *max* (B), *A*. *thaliana* (C), *B*. *rapa* (D), *N*. *tabacum* (E), *O*. *sativa* (F), *Z*. *mays* (G), *G*. *biloba* (H), and *P*. *patens* (I) were included in the analysis.
(PDF)

S10 Fig WGS, RNA-seq mapping and PCR amplicon sequencing at *rps2* gene. The alignments of WGS (A) and RNA-seq (B) were carried out by bowtie 2 and TopHat2, respectively. PCR amplicon (C) generated from high fidelity *Pfu* was directly sequenced using ABI PRISM 3730xl.
(PDF)

S11 Fig Sequence chromatograms of *rps2* RT-PCR amplicons. *V*. *radiata* (A), *G*. *max* (B), *A*. *thaliana* (C), *B*. *rapa* (D) and *N*. *tabacum* (E) were included in the analysis.
(PDF)

S1 Table Primers used in this study. (DOC)

S2 Table Read statistics, error correction and trimming for gDNA samples (DOCX)

S3 Table Distribution of different repeat types. (DOC)

S4 Table Summary of single nucleotide variations in *V*. *radiata* var. KPS1 and NM92 with TC1966 used as a reference. (DOC)

S5 Table Summary of indels in *V*. *radiata* var. KPS1 and NM92 with TC1966 used as a reference. (DOC)

S6 Table SSR loci and TS frequencies. (DOC)

S7 Table SRA data used in this study. (DOC)
