# Supplementary figures and images for: Transcriptional Slippage and RNA Editing Increase the Diversity of Transcripts in Chloroplasts: Insight from Deep Sequencing of Vigna radiata Genome and Transcriptome
Source: PLoS One. 2015 Jun 15;10(6):e0129396. doi: 10.1371/journal.pone.0129396 (PMC4468118; doi:10.1371/journal.pone.0129396)

S1 Fig.

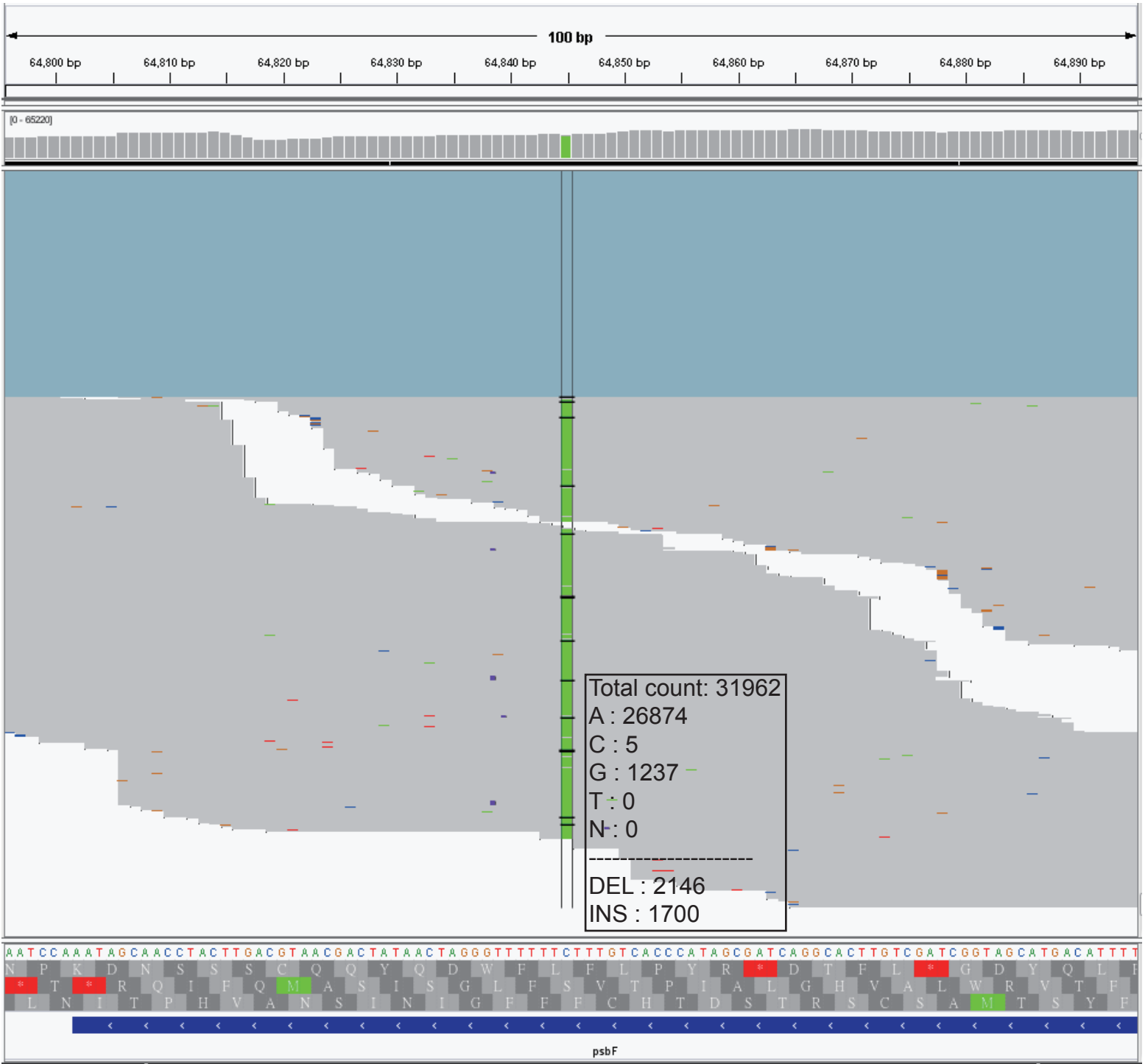

Supplement: S1 Fig — The editing site is flanked by two vertical black lines. The read sequence at the flanked site is calculated and shown in the black square. The horizontal black line indicates deletions in a read, and the purple spots show insertions in overlapping reads. (PDF) [file pone.0129396.s001.pdf]

S3 Fig.

(A) WGS reads

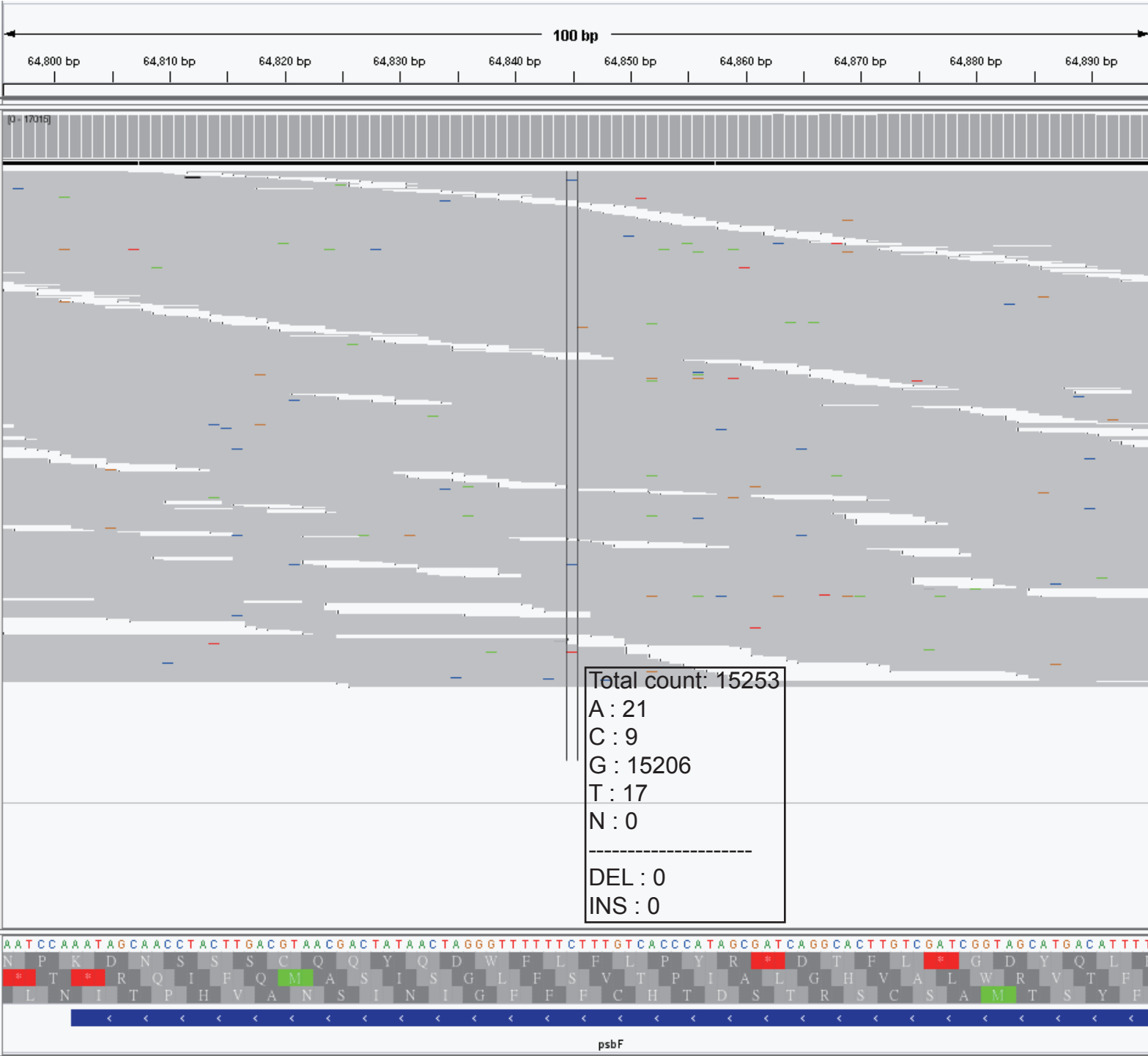

(B) RNA-seq reads

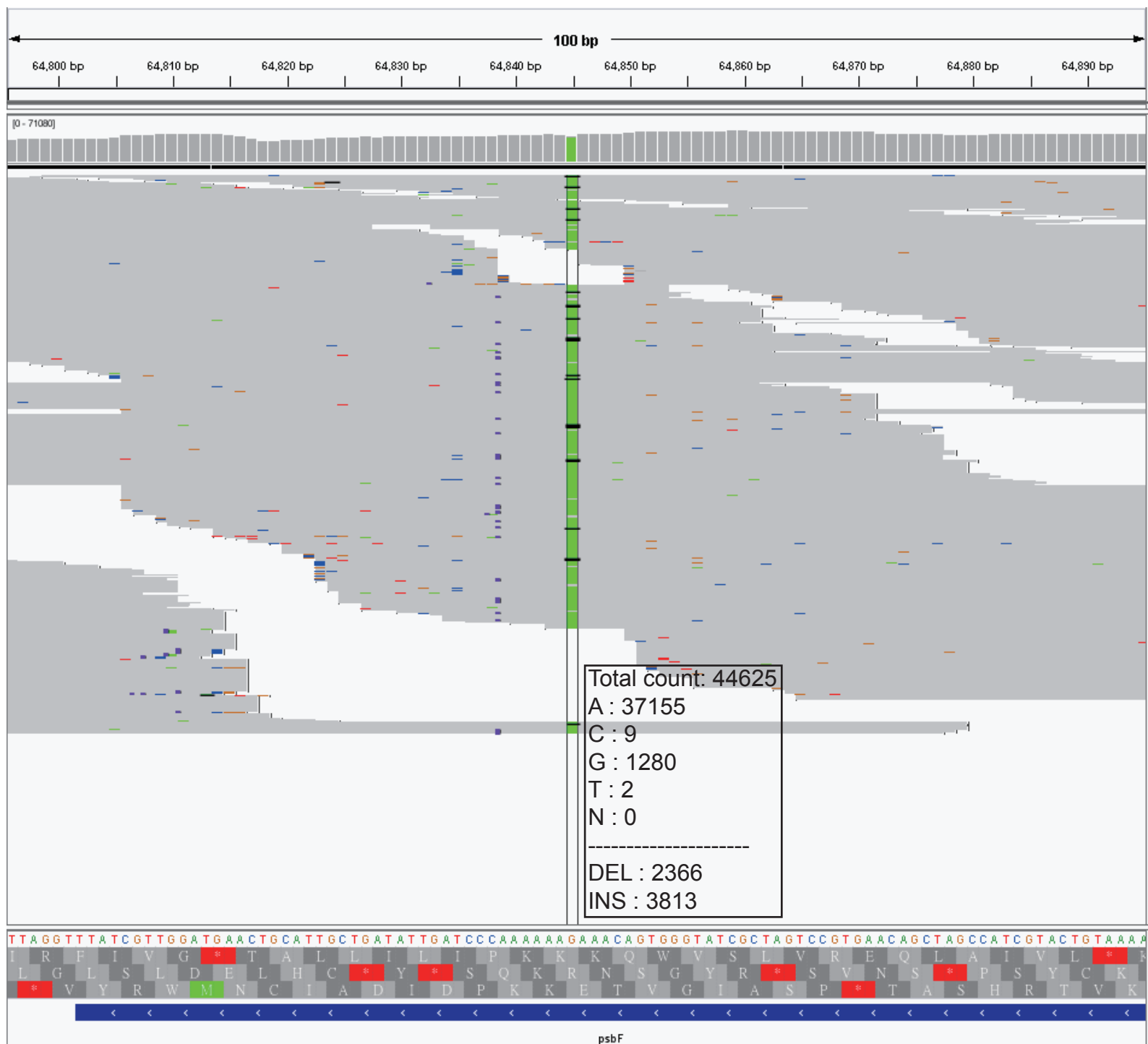

Supplement: S3 Fig — WGS (A) and RNA-seq (B) reads were mapped to the psbF gene. The editing site is flanked by two vertical dashed lines. The read sequence at the flanked site is calculated and shown in the black square. The horizontal black line indicates deletions in a read, and the purple spots show insertions in overlapping reads. (PDF) [file pone.0129396.s003.pdf]

S4 Fig.

(A) GM

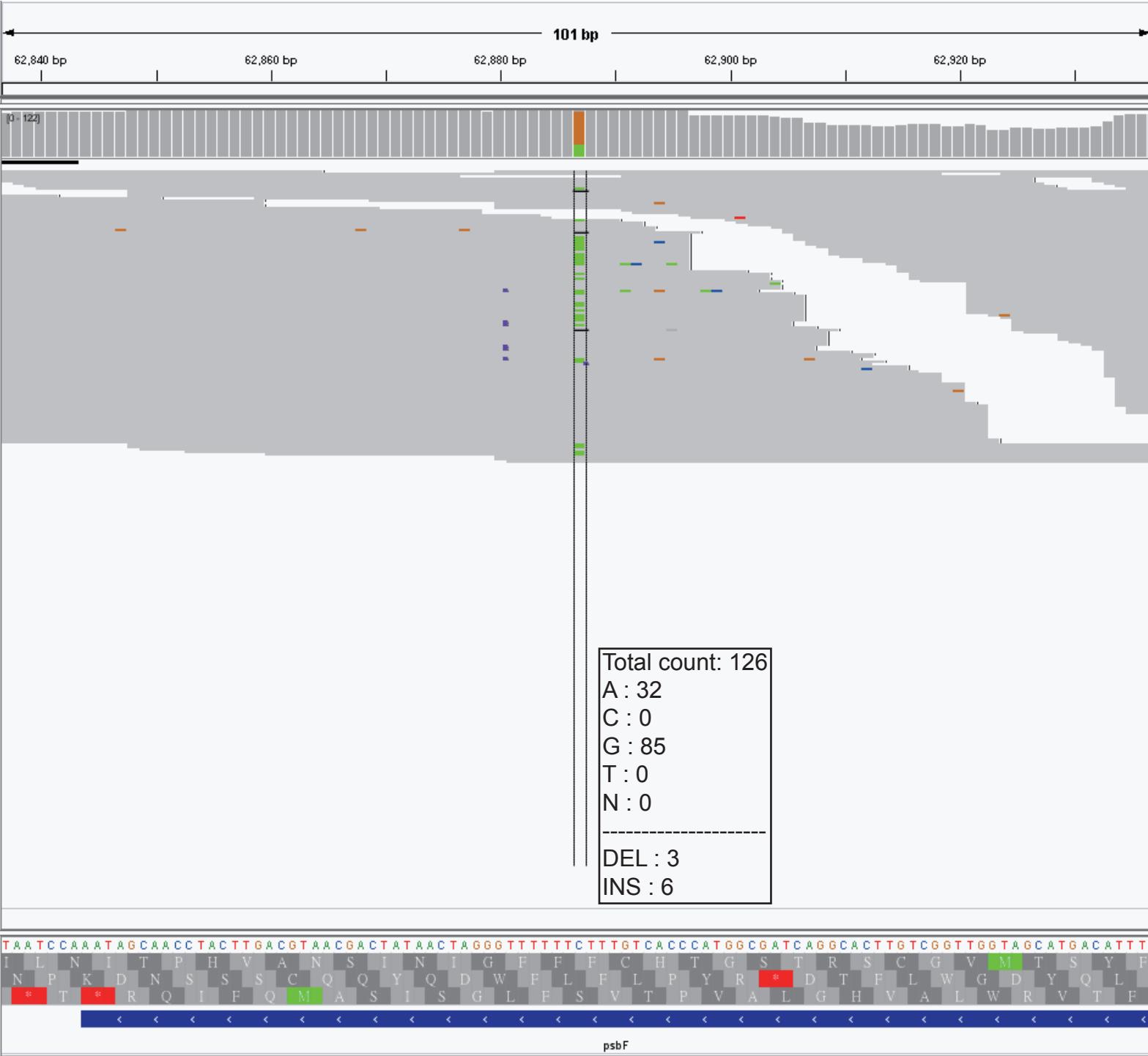

S4 Fig.

(B) AT

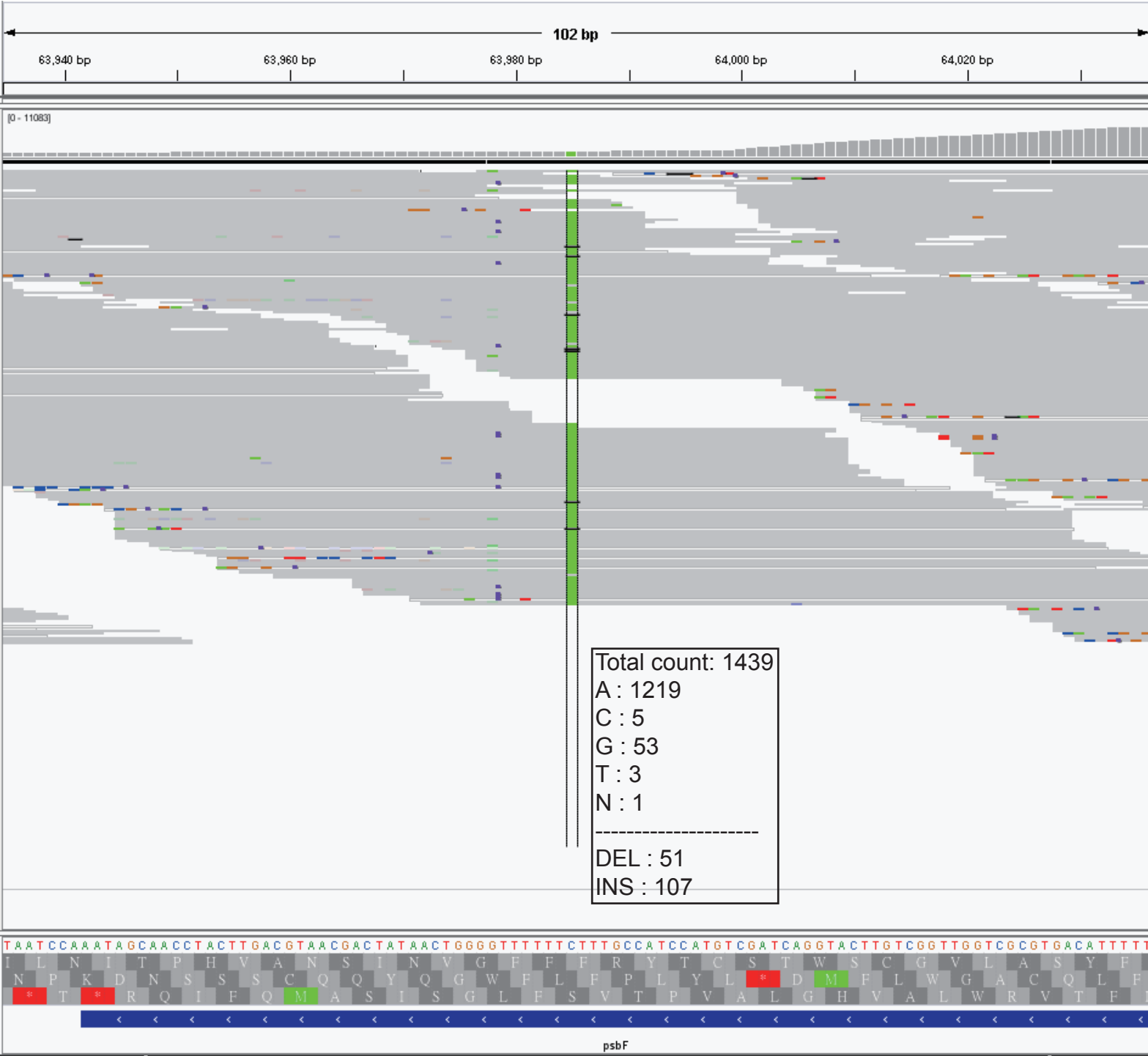

S4 Fig.

(C) BR

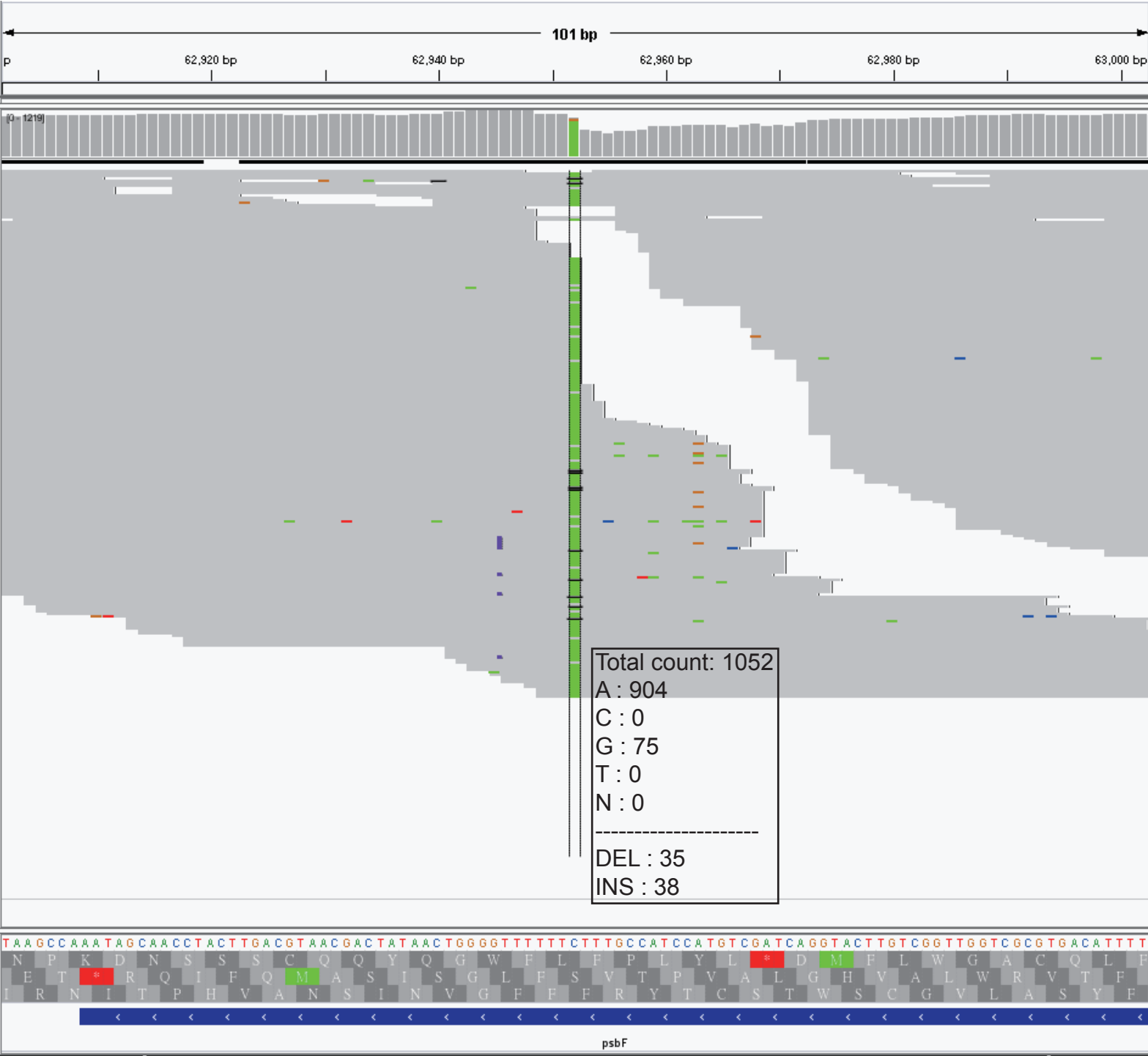

S4 Fig.

(D) NT

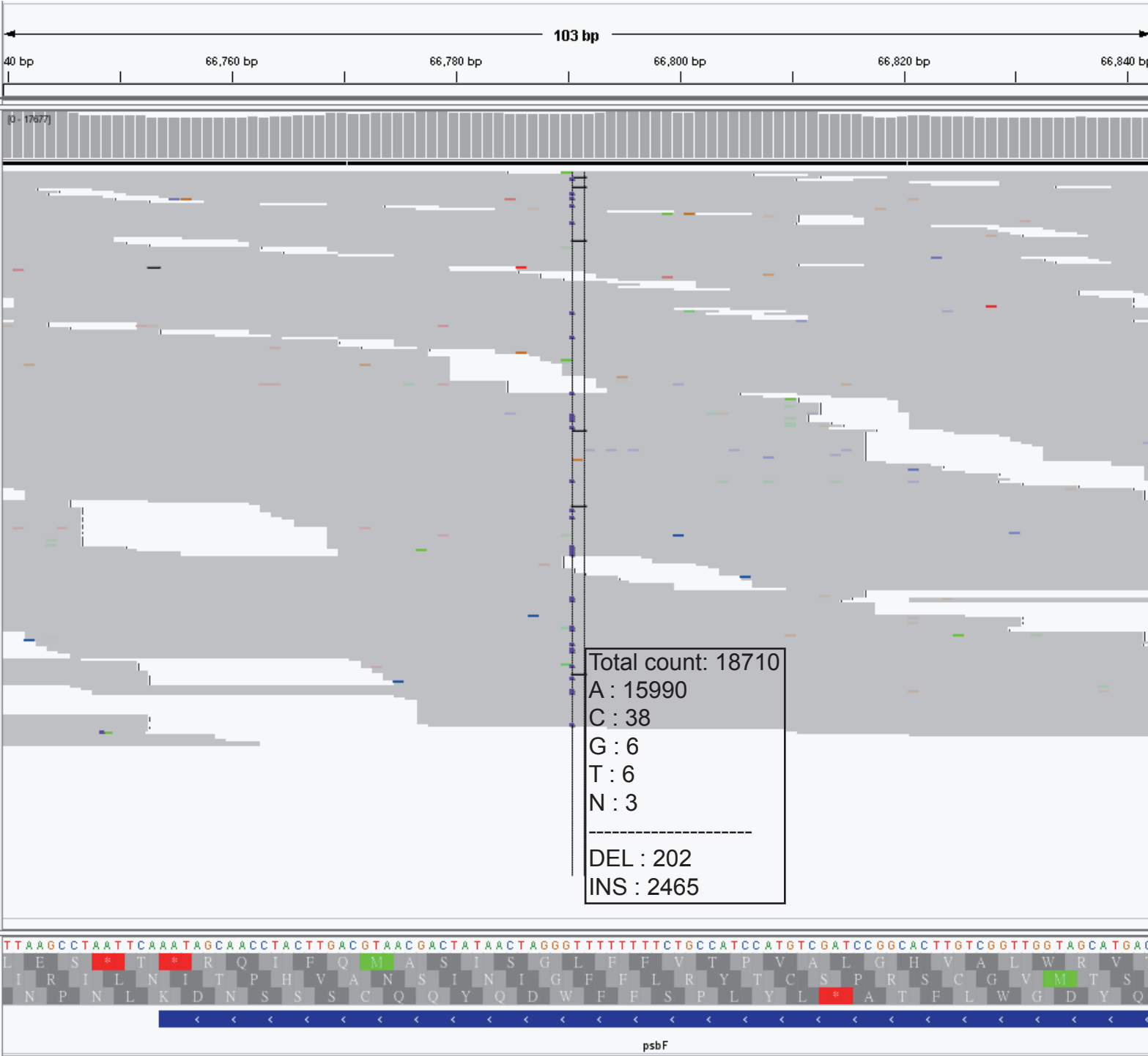

S4 Fig.

(E) OS

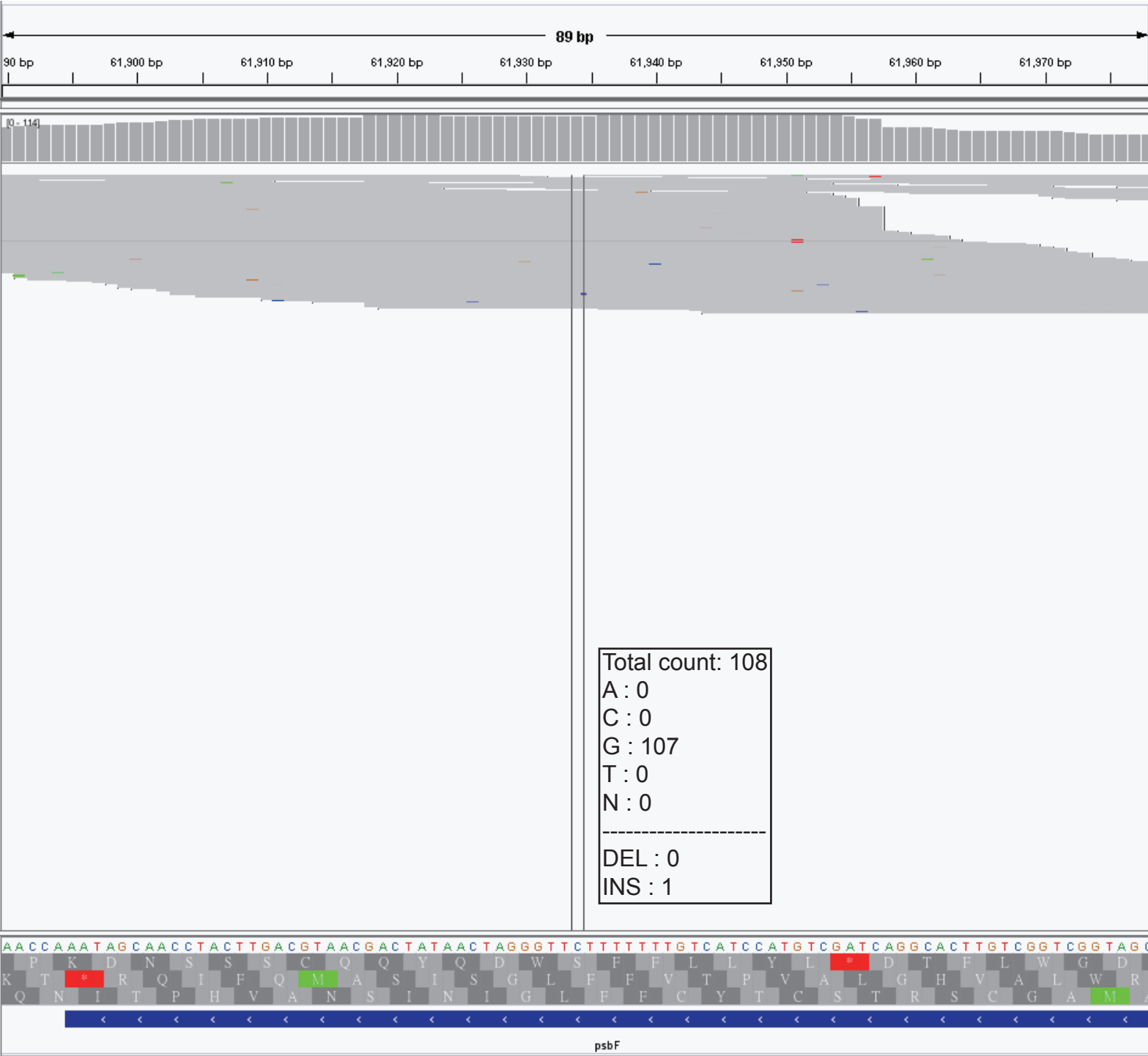

S4 Fig.

(F) ZM

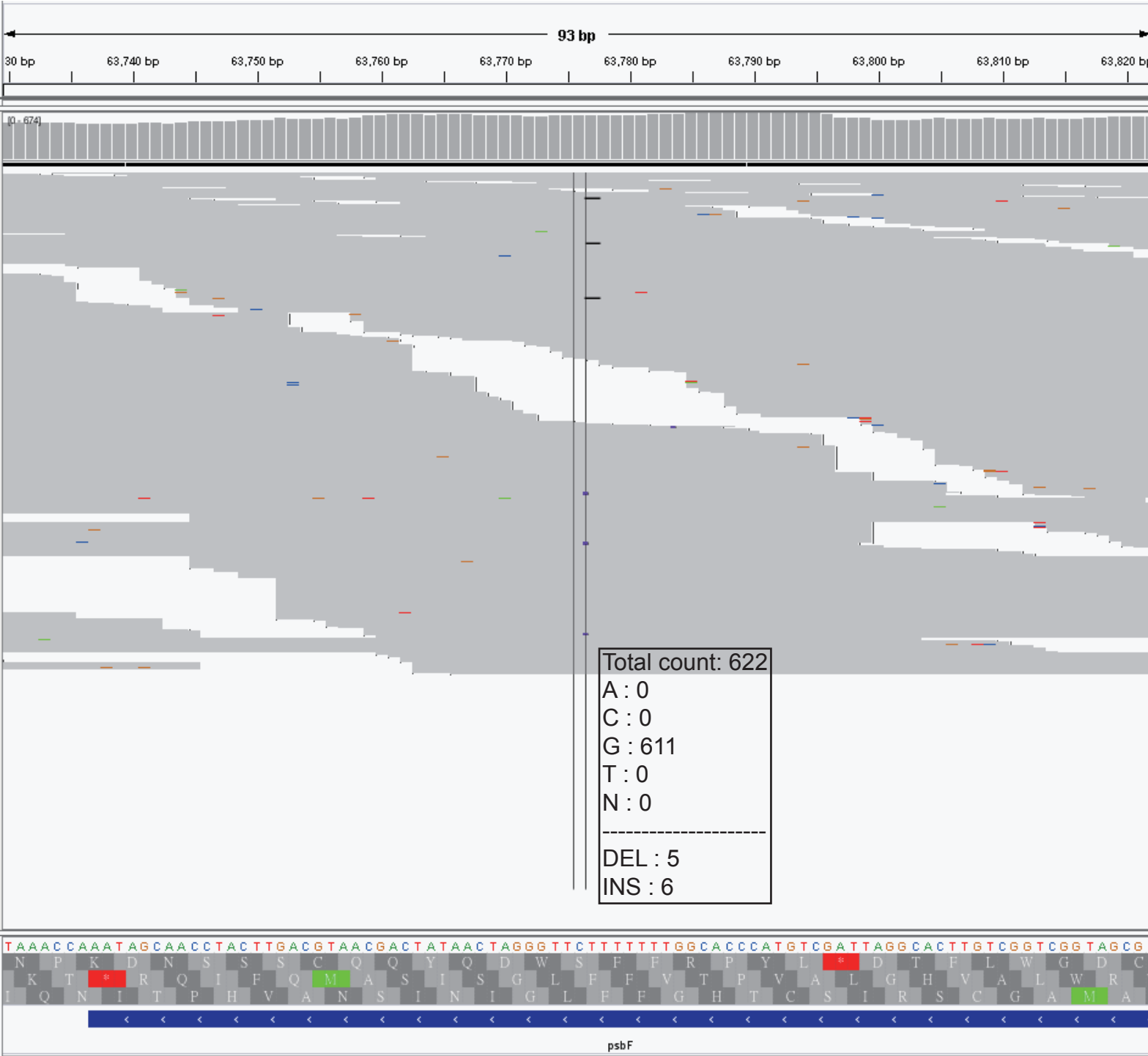

S4 Fig.

(G) PT

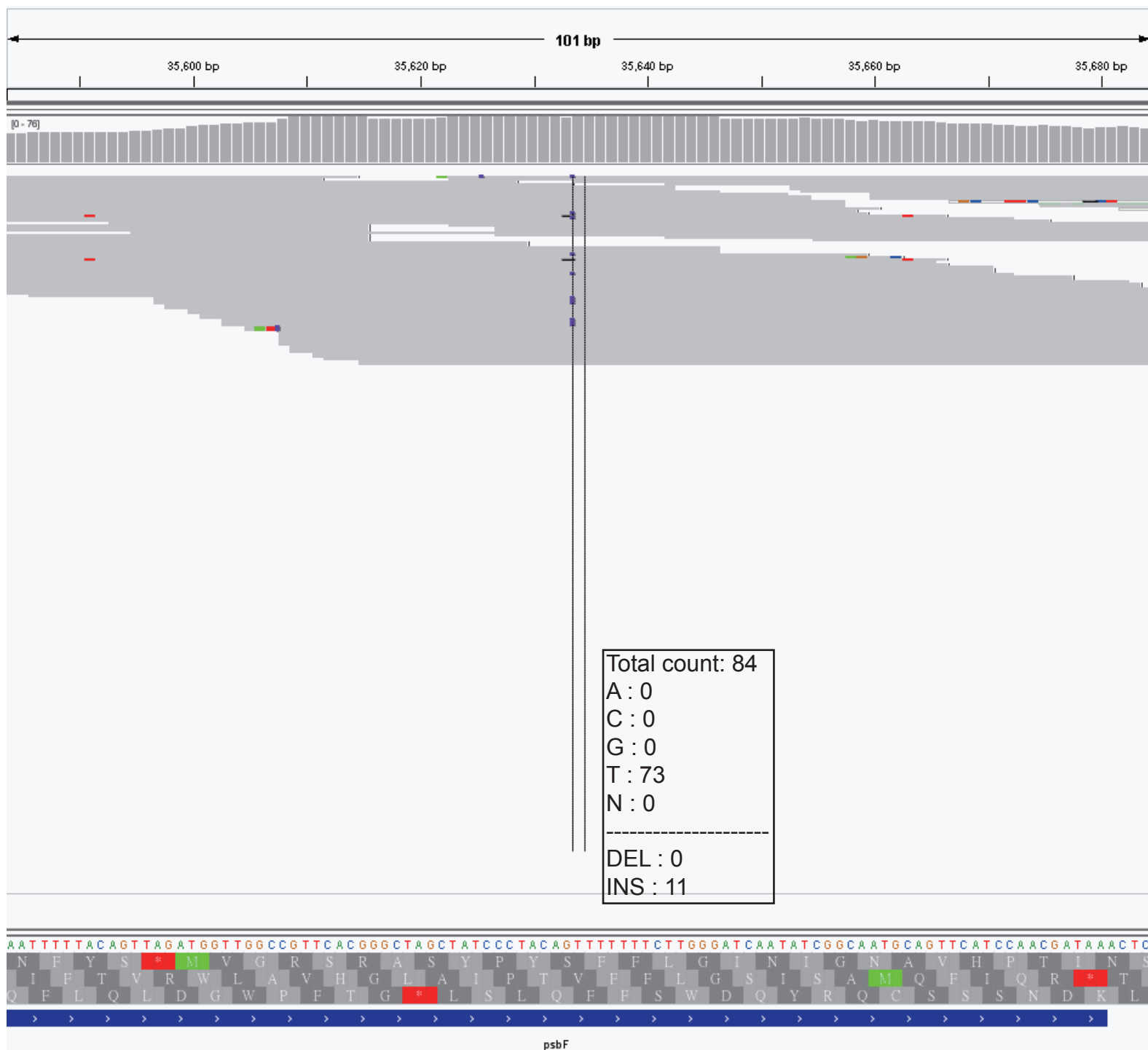

S4 Fig.

(H) GB

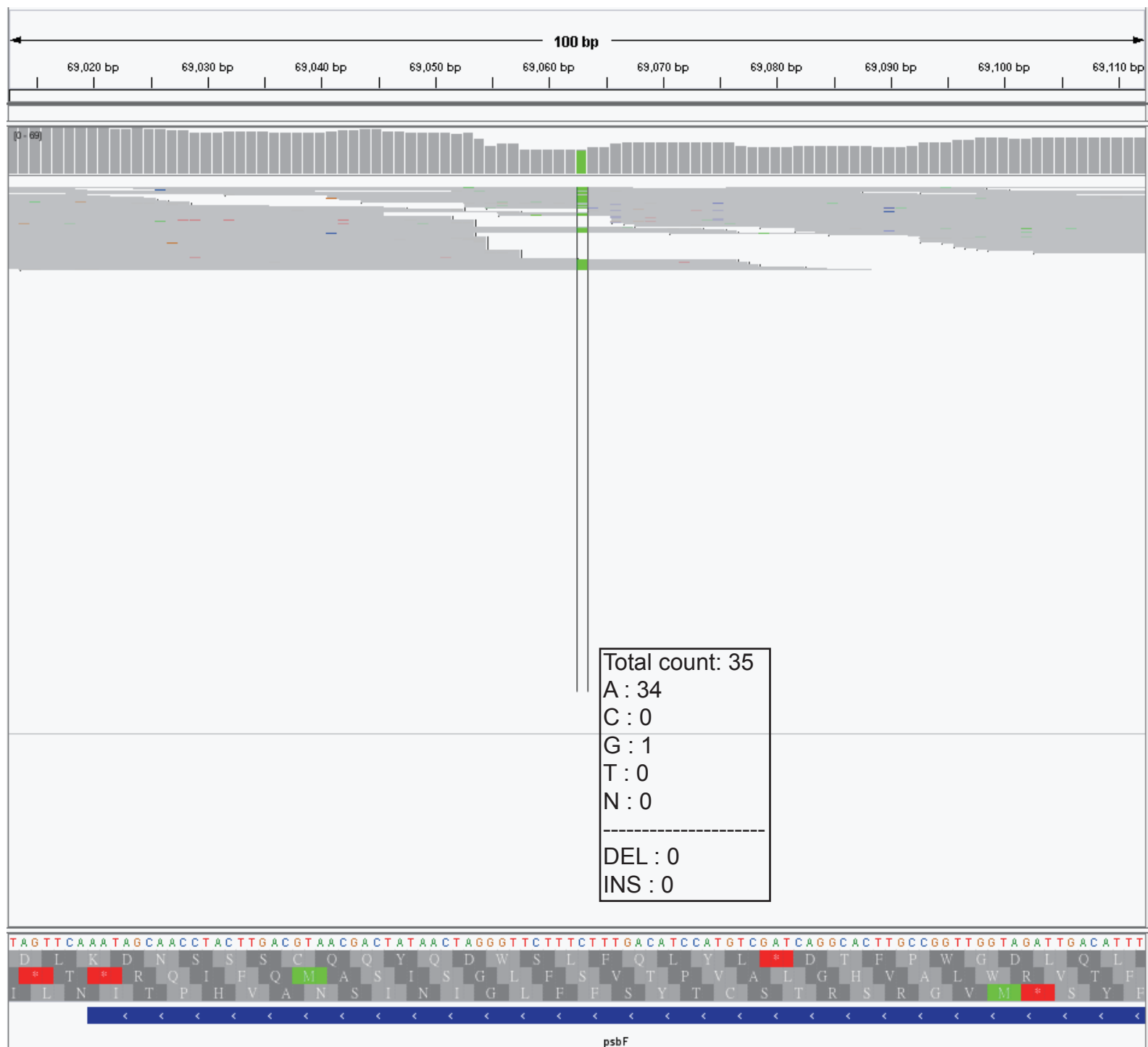

(I) PP

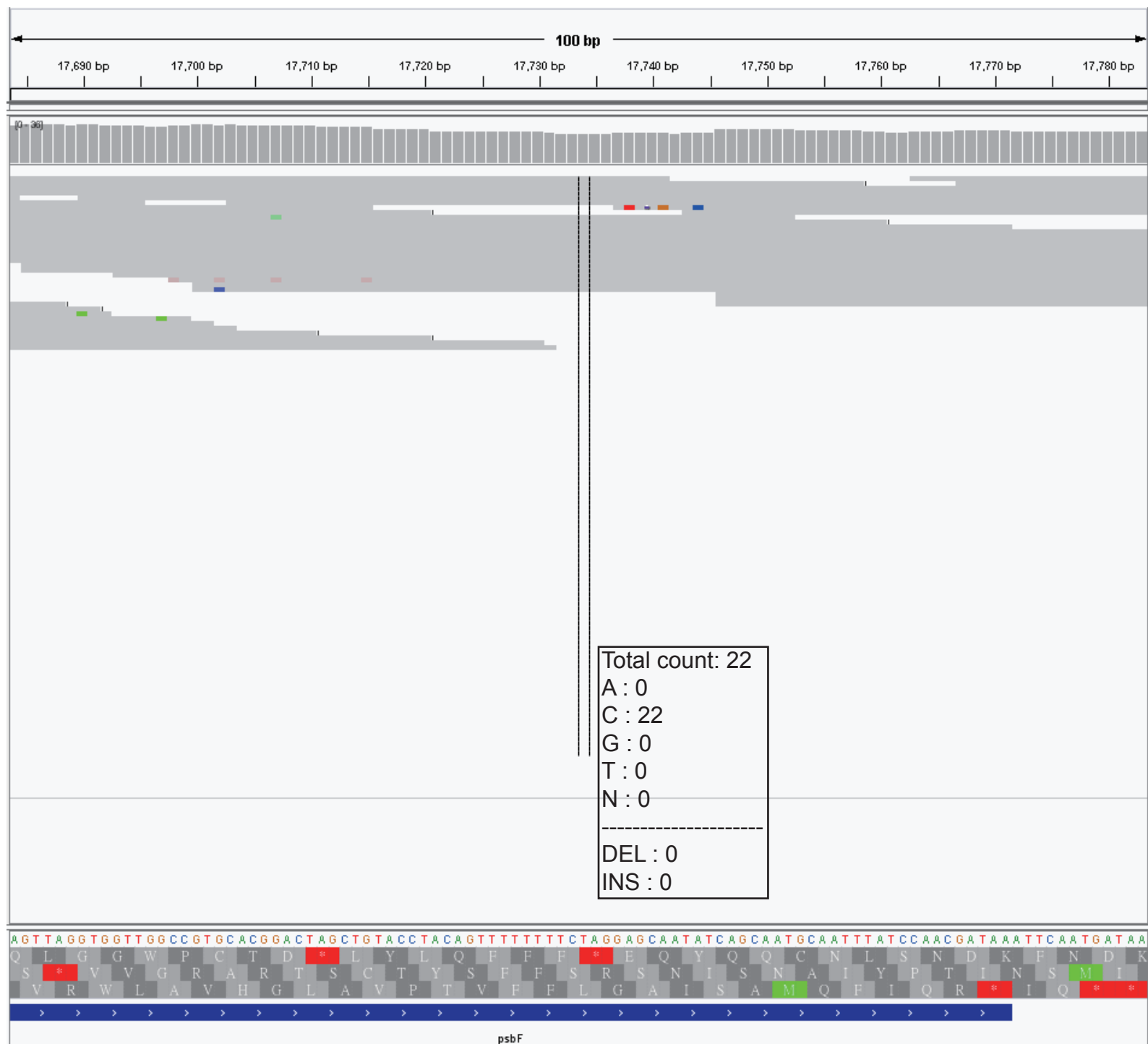

Supplement: S4 Fig — G. max (A), A. thaliana (B), B. rapa (C), N. tabacum (D), O. sativa (E), Z. mays (F), P. taeda (G), G. biloba (H), and P. patens (I) were included in the analysis. The horizontal black line indicates a deletion, and the purple spot indicates an insertion. The black square denotes the count of read sequences at the site flanked by two vertical dashed lines. (PDF) [file pone.0129396.s004.pdf]

(A) normal transcript

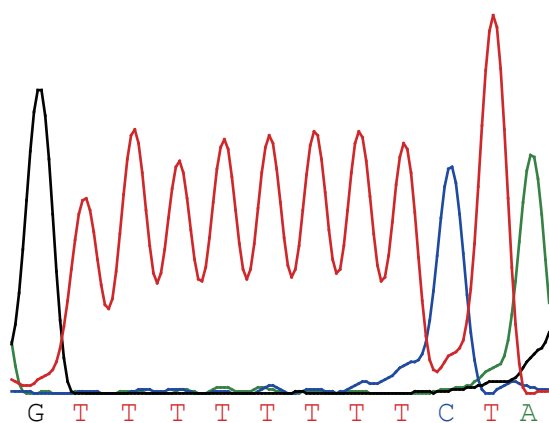

(B) U-deletion transcript

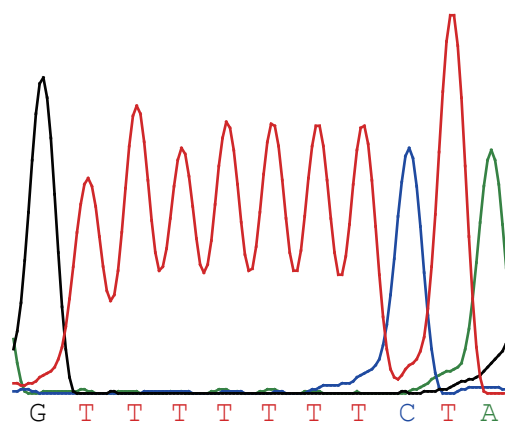

(C) U-insertion transcript

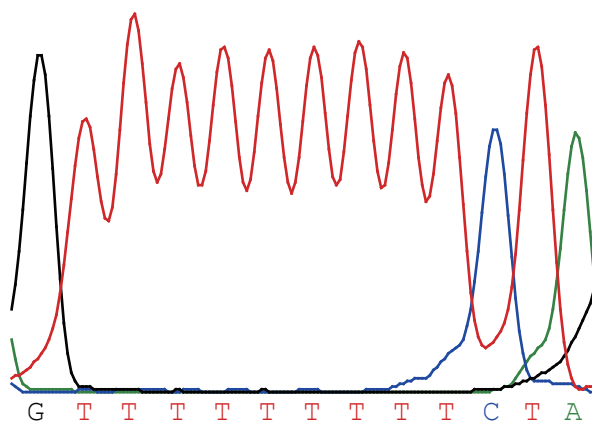

Supplement: S6 Fig — Three kinds of sequences were identified: normal transcripts (A), U-deletion transcripts (B) and U-insertion transcripts (C). (PDF) [file pone.0129396.s006.pdf]

S7 Fig.

(A) WGS reads

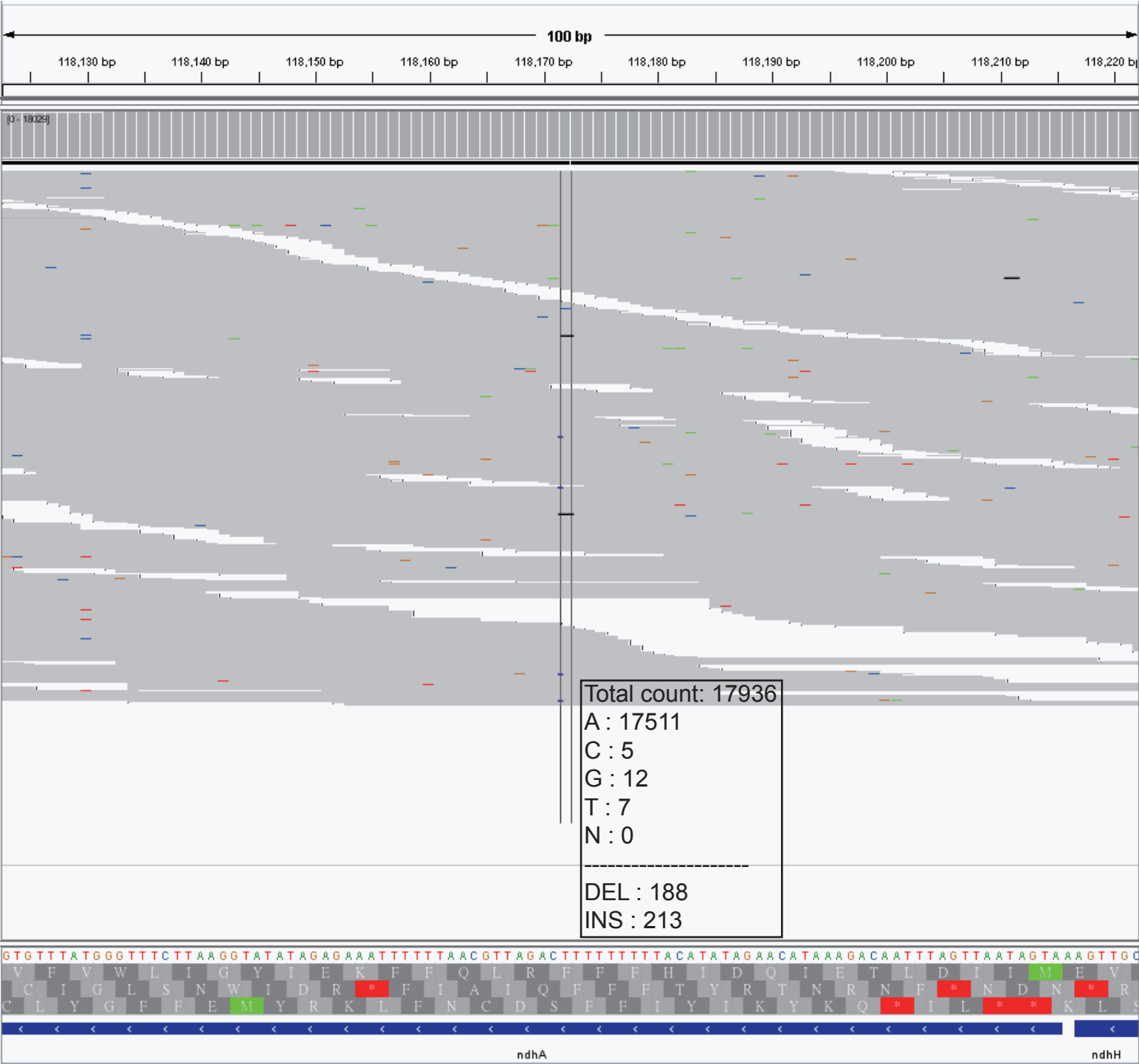

S7 Fig.

(B) RNA-seq reads

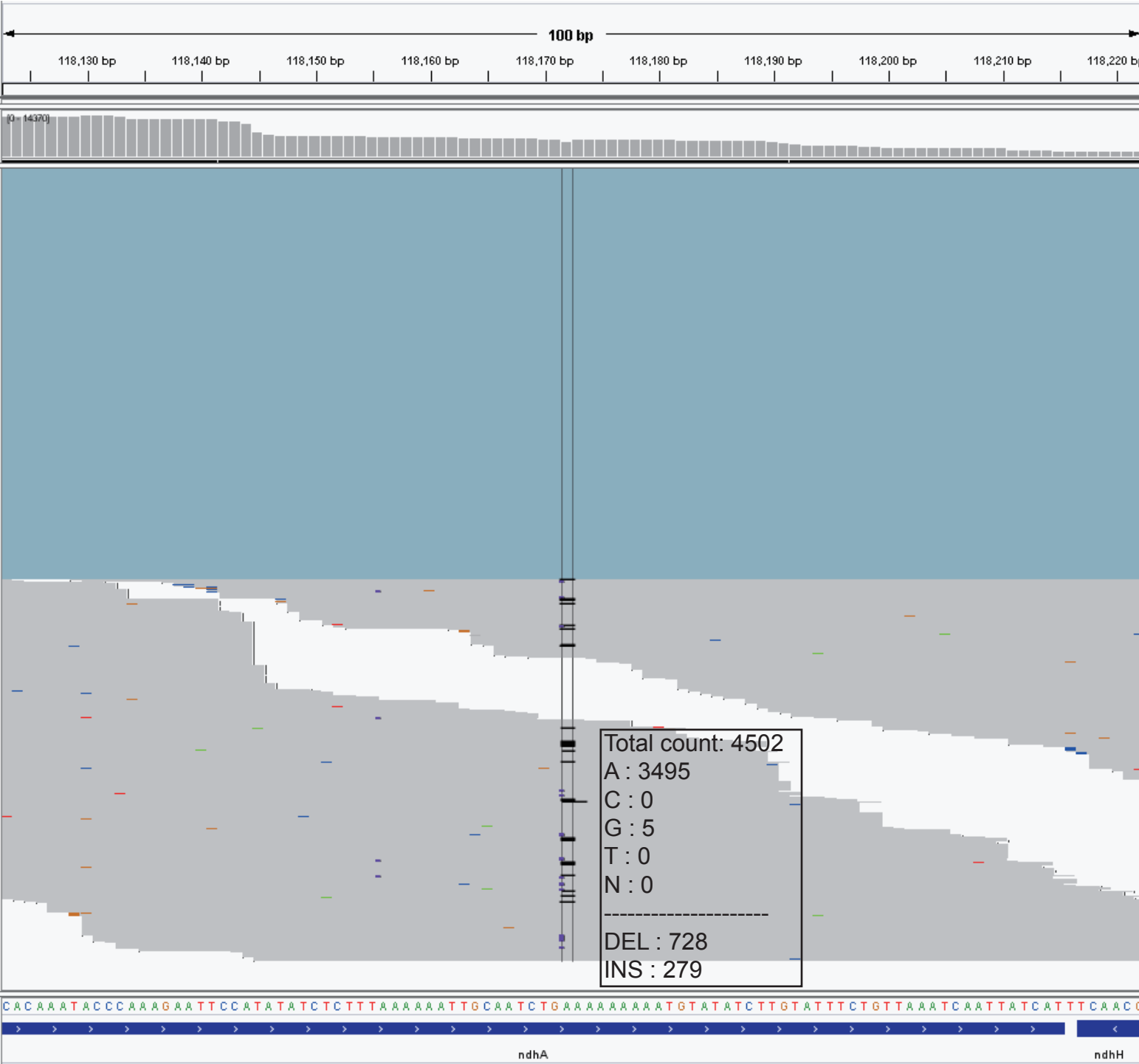

(C) PCR amplicon

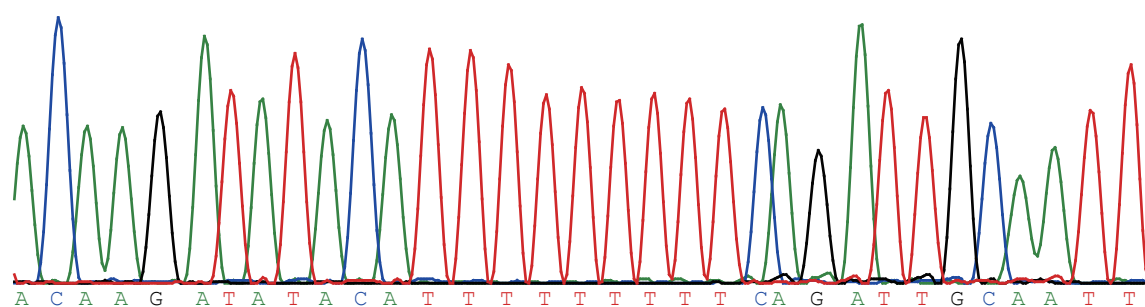

Supplement: S7 Fig — The alignments of WGS (A) and RNA-seq (B) were carried out by bowtie 2 and TopHat2, respectively. PCR amplicon (C) generated from high fidelity Pfu was directly sequenced using ABI PRISM 3730xl. (PDF) [file pone.0129396.s007.pdf]

S8 Fig.

(A) VR

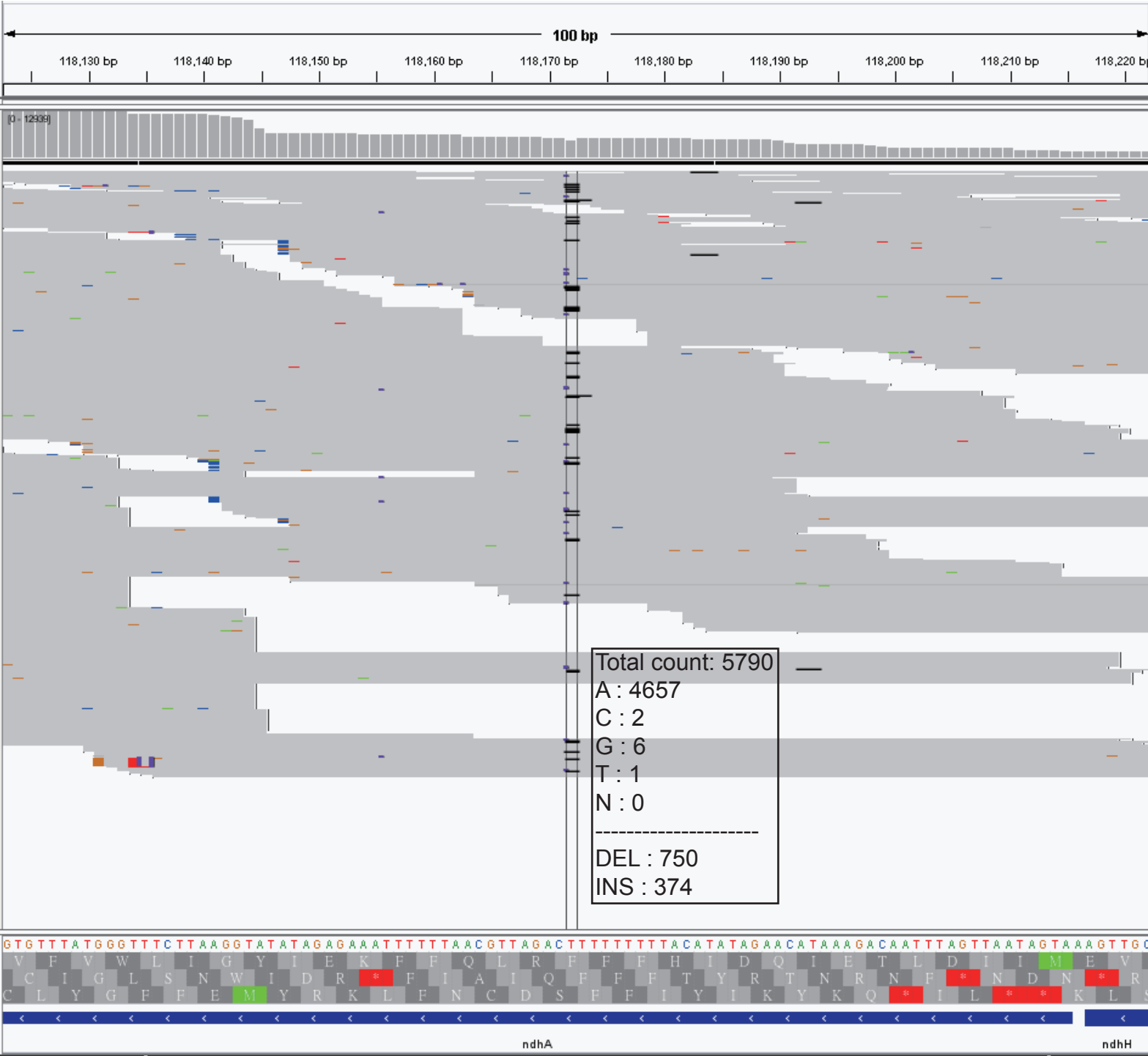

(B) GM

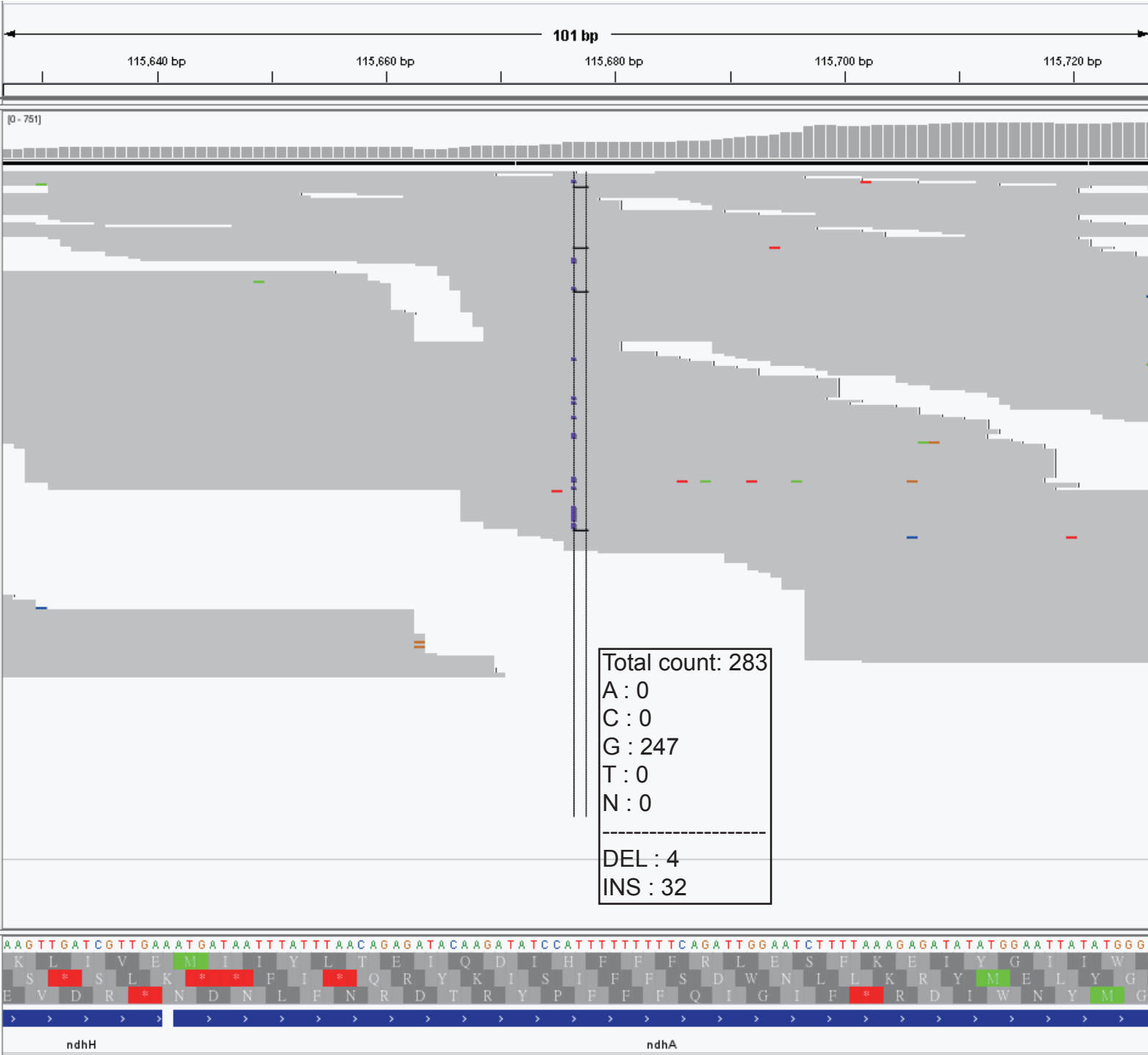

S8 Fig.

(C) AT

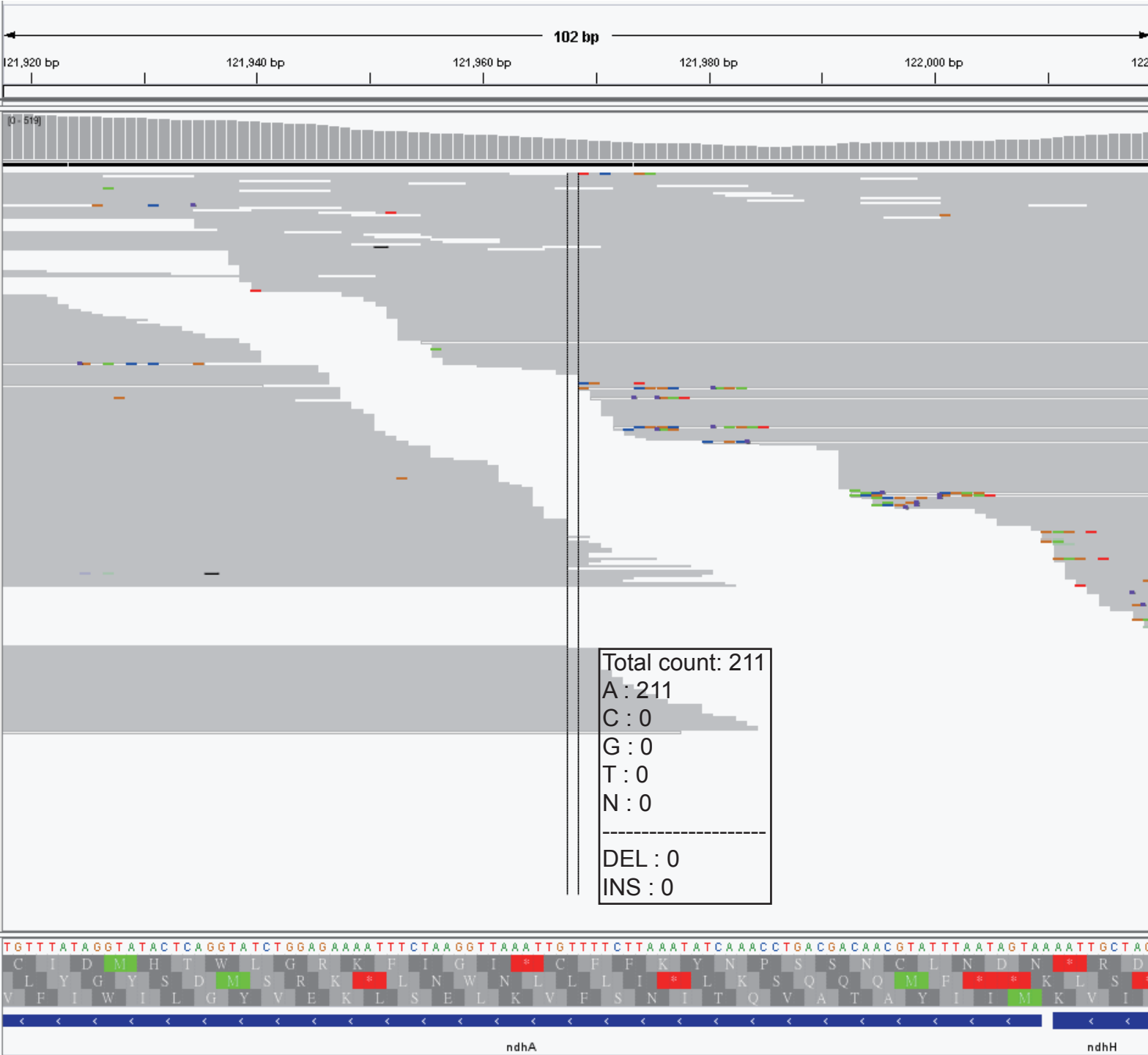

S8 Fig.

(D) BR

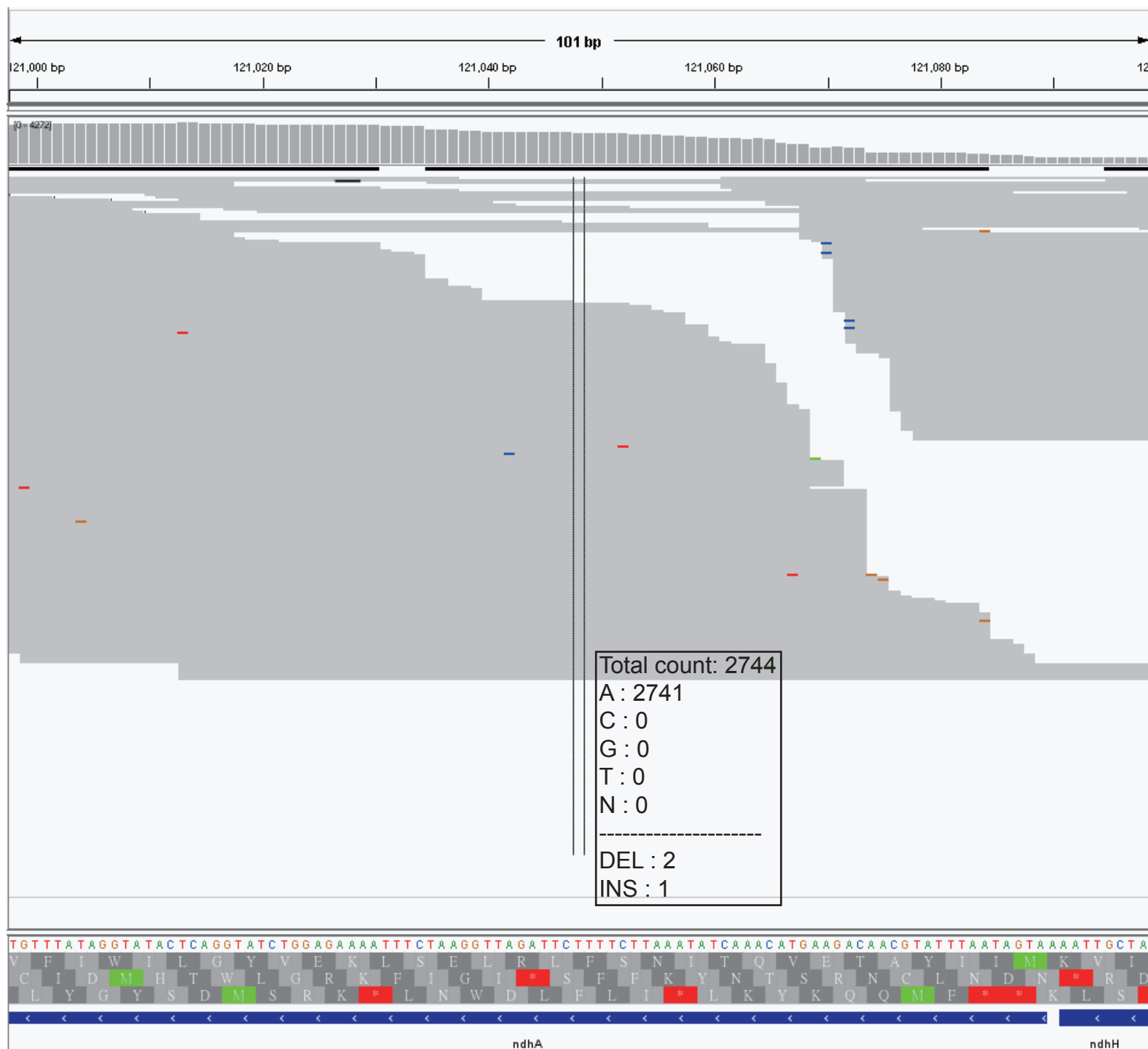

S8 Fig.

(E) NT

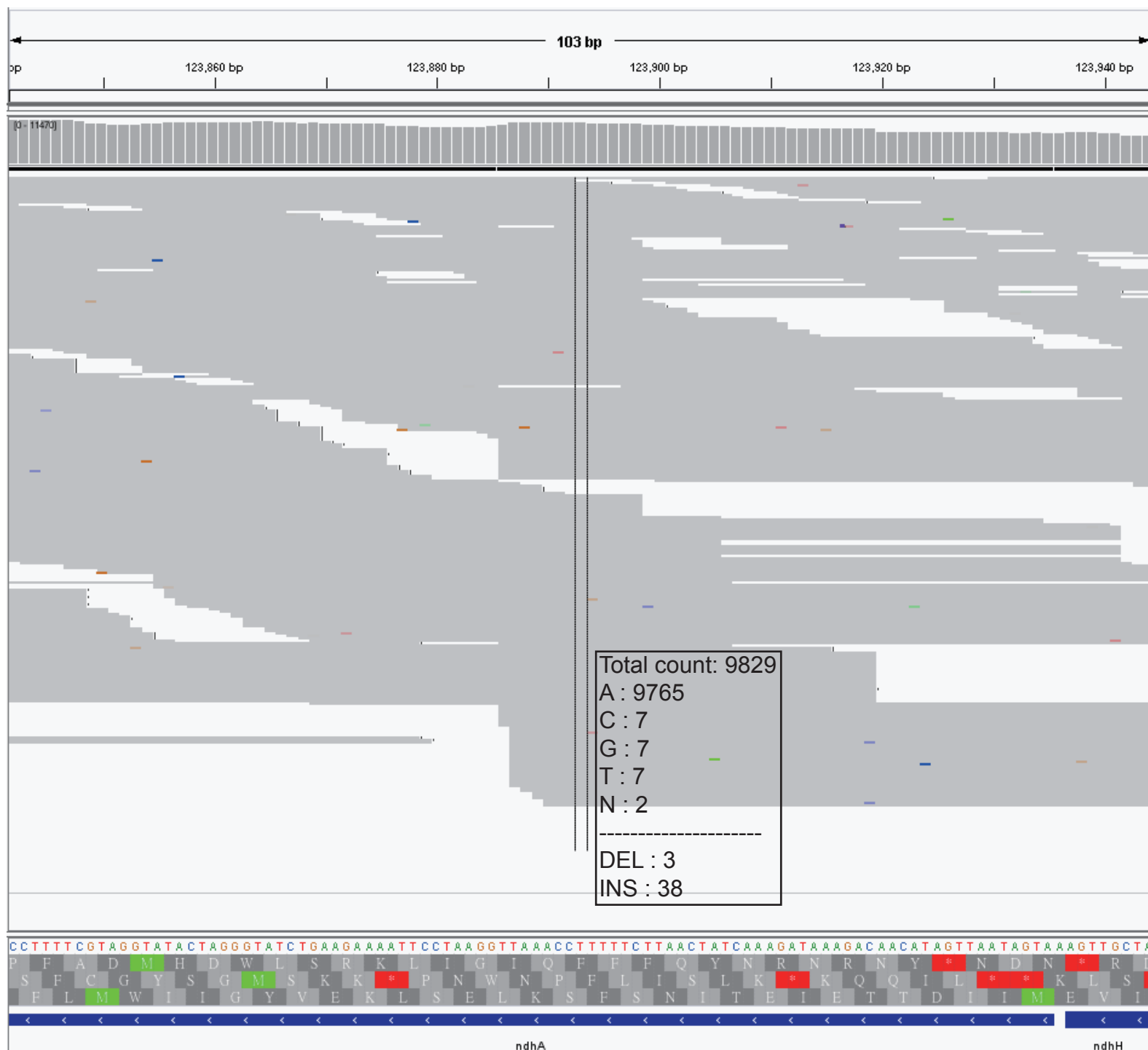

S8 Fig.

(F) OS

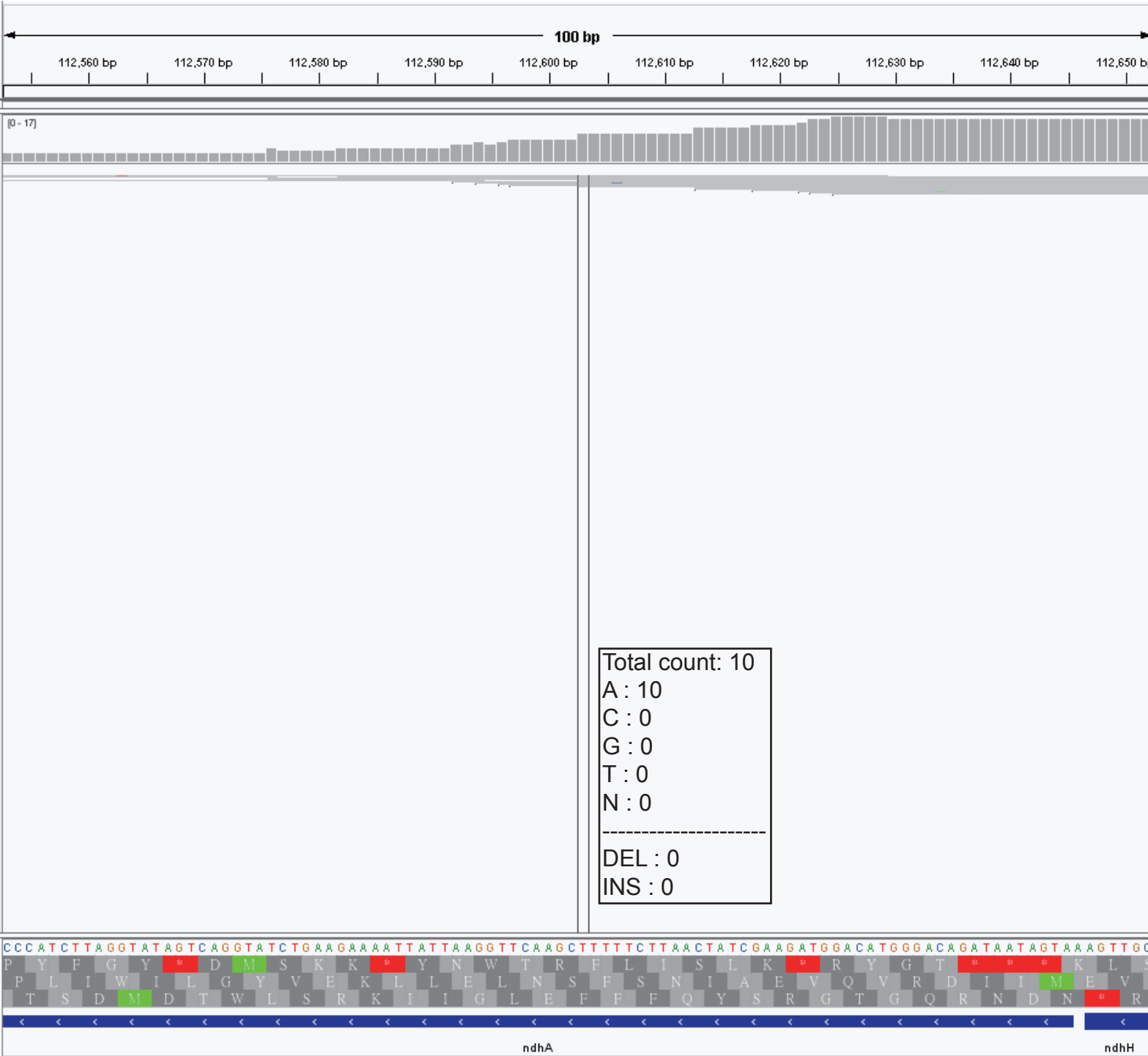

(G) ZM

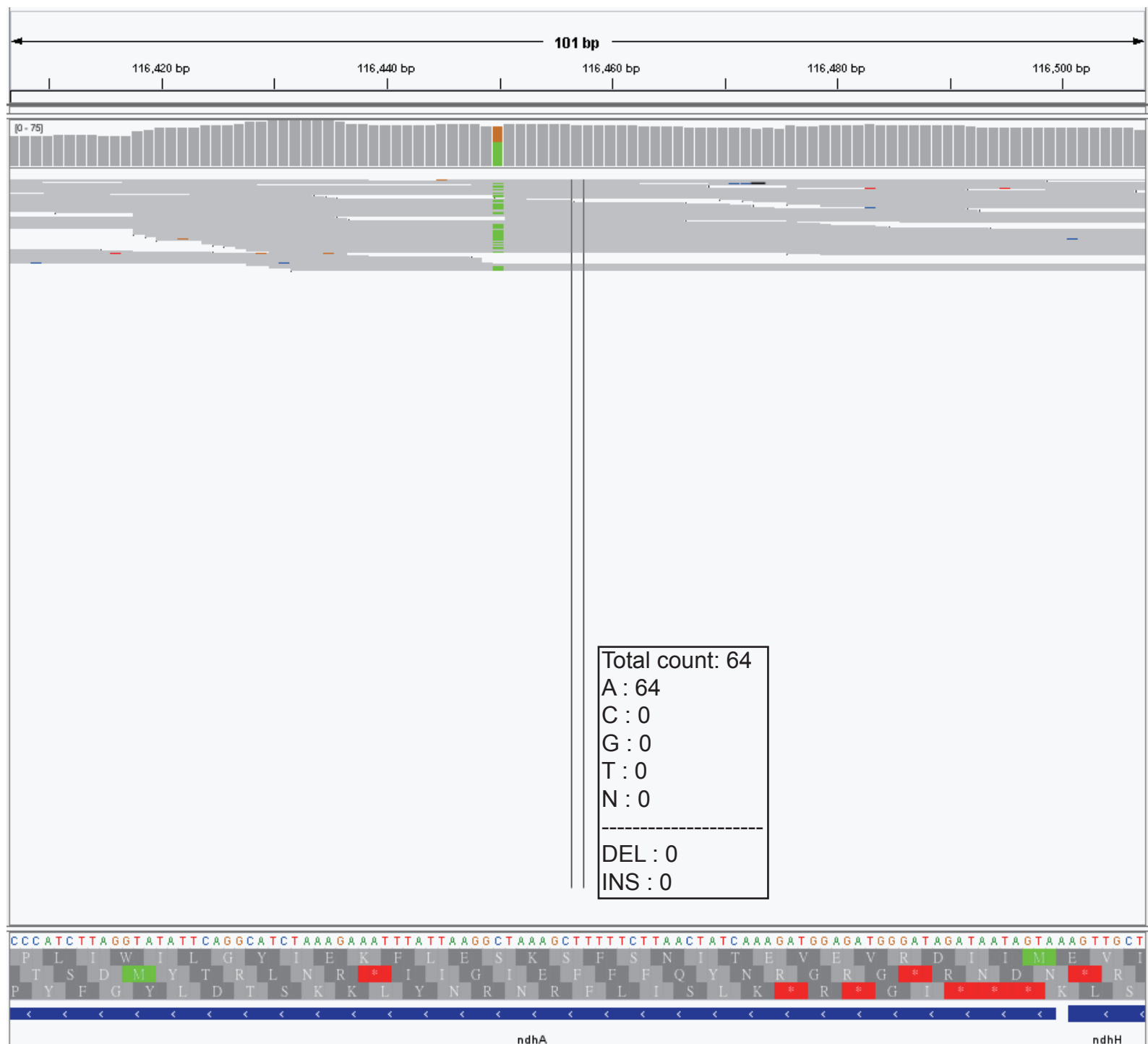

S8 Fig.

(H) GB

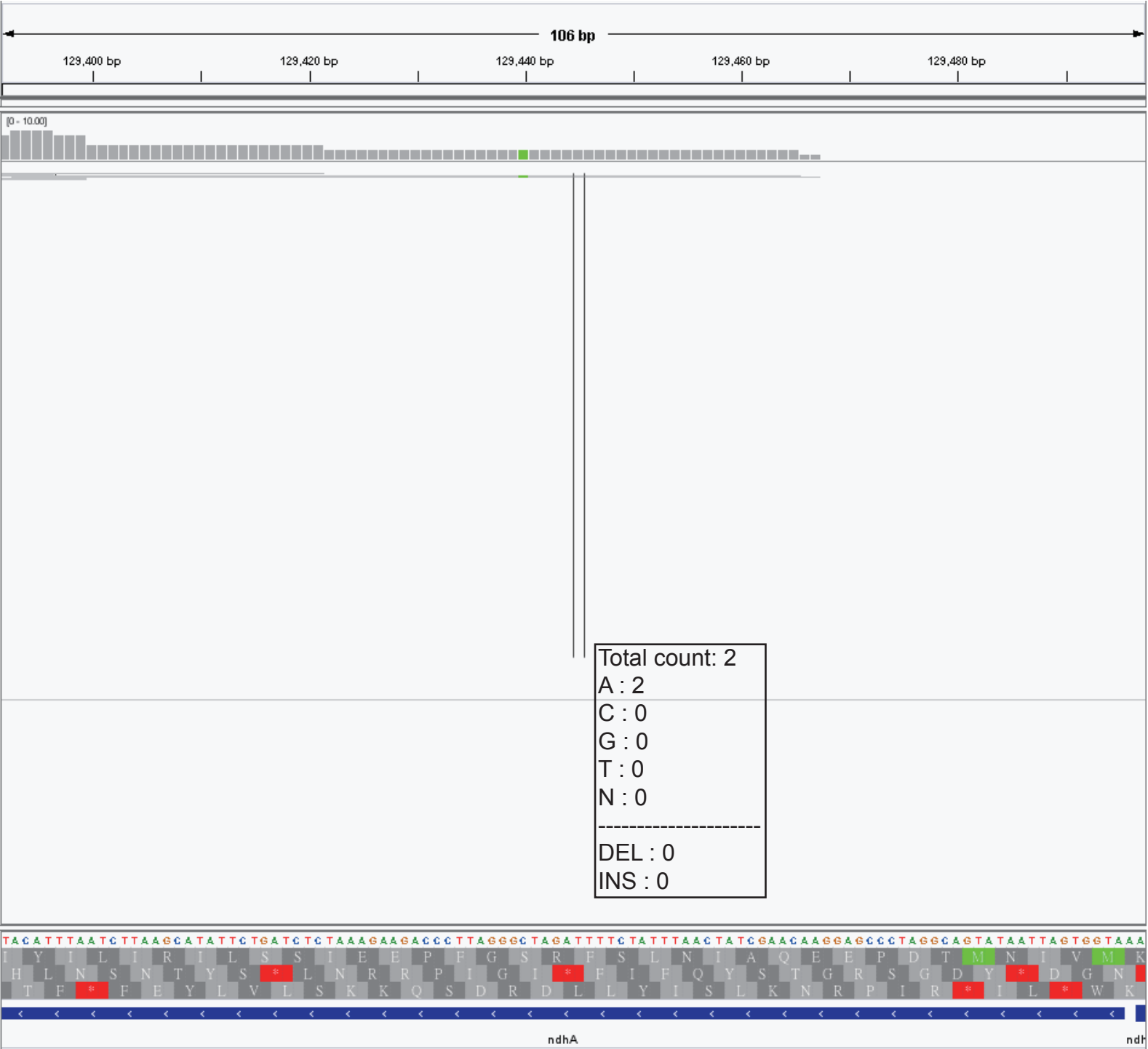

S8 Fig.

(I) PP

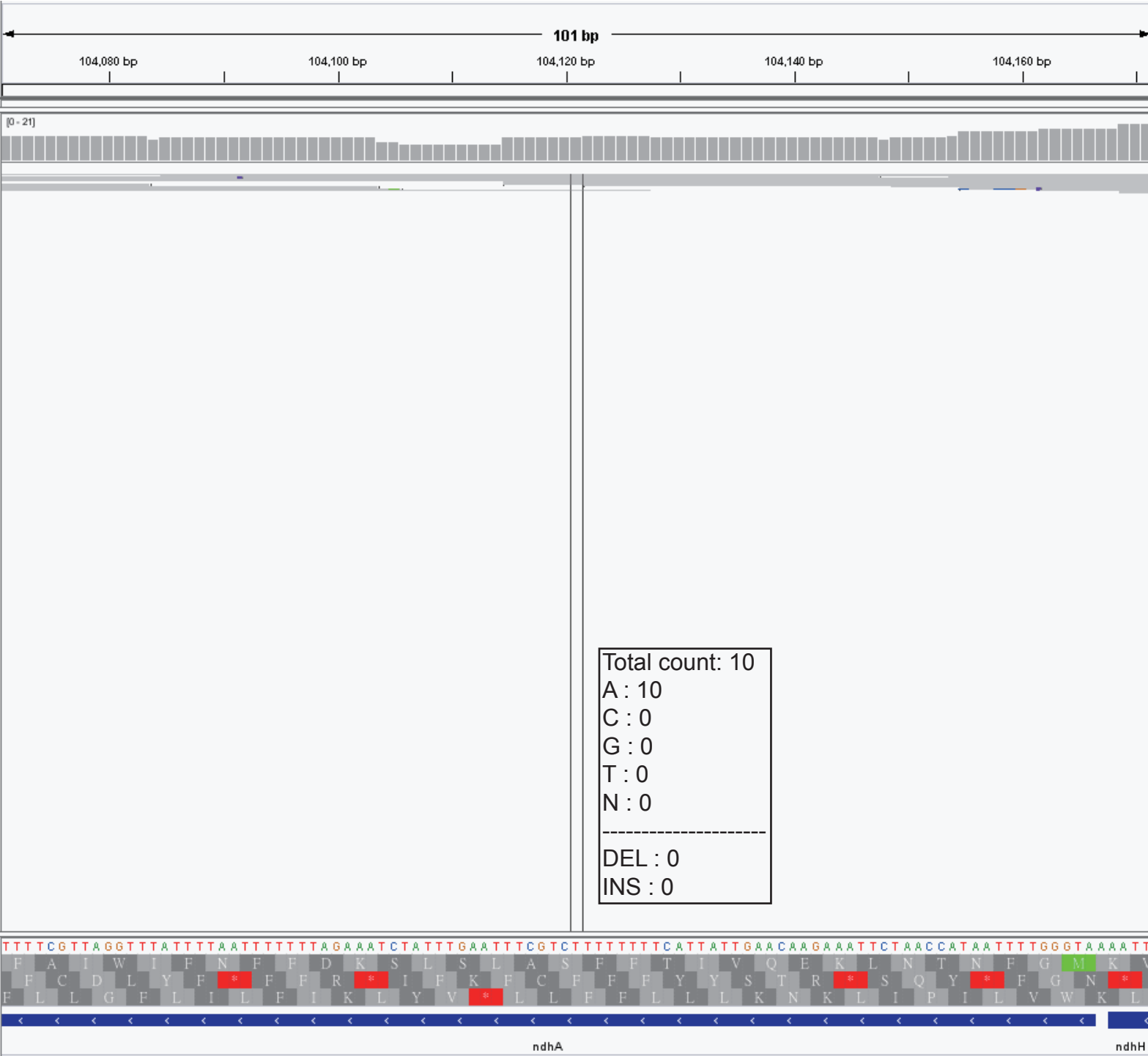

Supplement: S8 Fig — V. radiata (A), G. max (B), A. thaliana (C), B. rapa (D), N. tabacum (E), O. sativa (F), Z. mays (G), G. biloba (H), and P. patens (I) were included in the analysis. The black line indicates a deletion, and the purple I character indicates an insertion. The black square denotes the count of read sequences at the site flanked by two vertical dashed lines. (PDF) [file pone.0129396.s008.pdf]

S9 Fig.

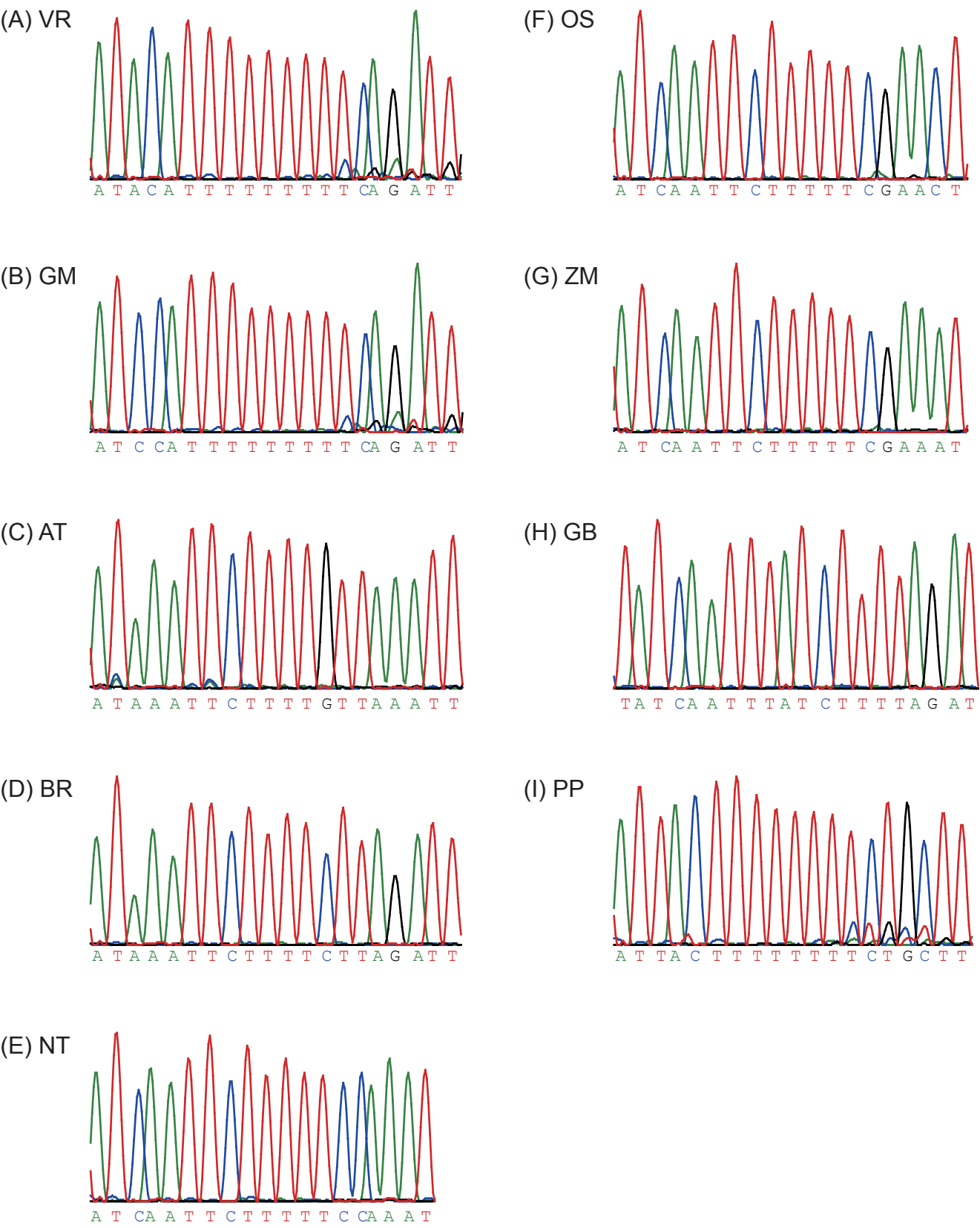

Supplement: S9 Fig — V. radiata (A), G. max (B), A. thaliana (C), B. rapa (D), N. tabacum (E), O. sativa (F), Z. mays (G), G. biloba (H), and P. patens (I) were included in the analysis. (PDF) [file pone.0129396.s009.pdf]

S10 Fig.

(A) WGS reads

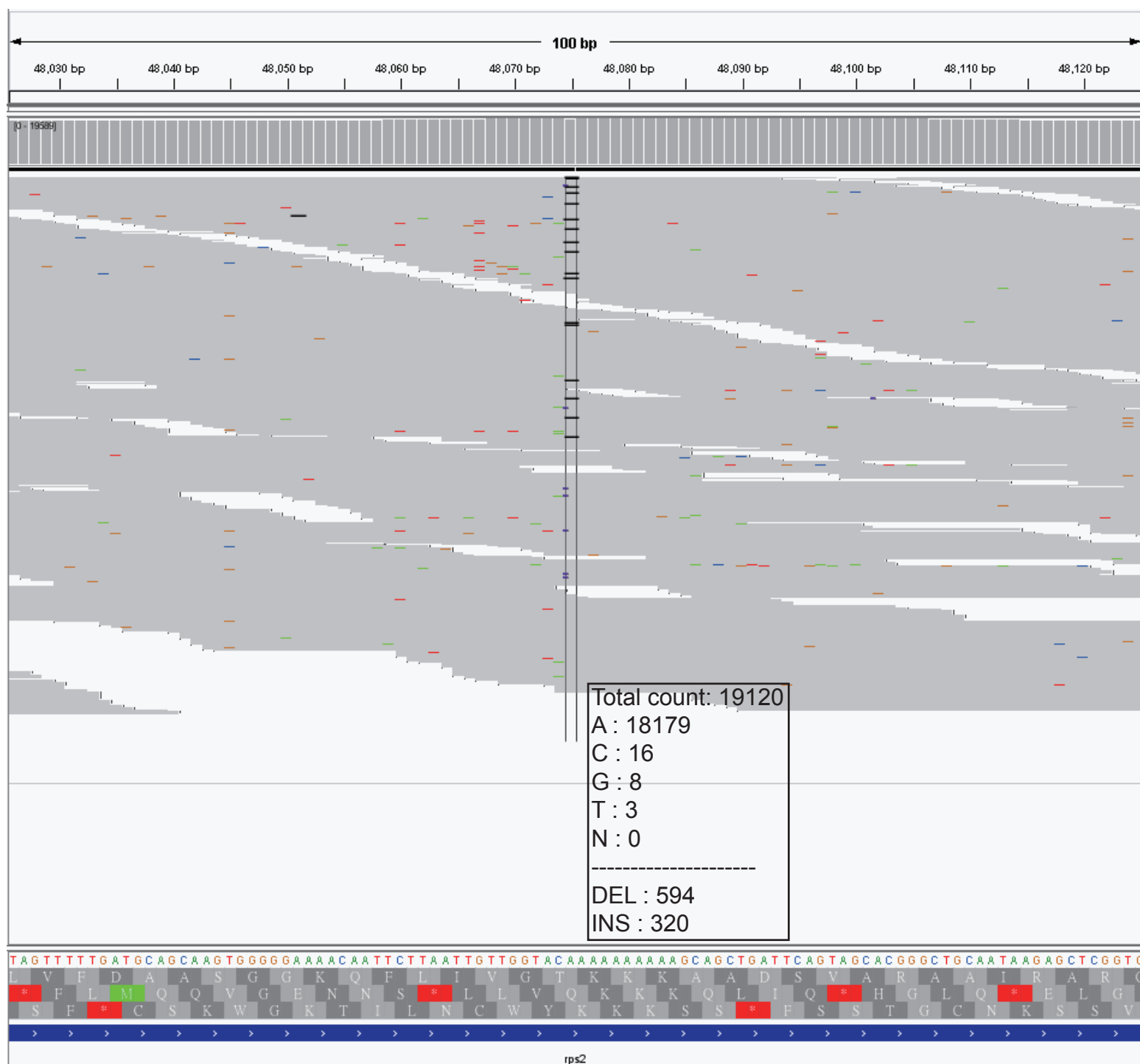

S10 Fig.

(B) RNA-seq reads

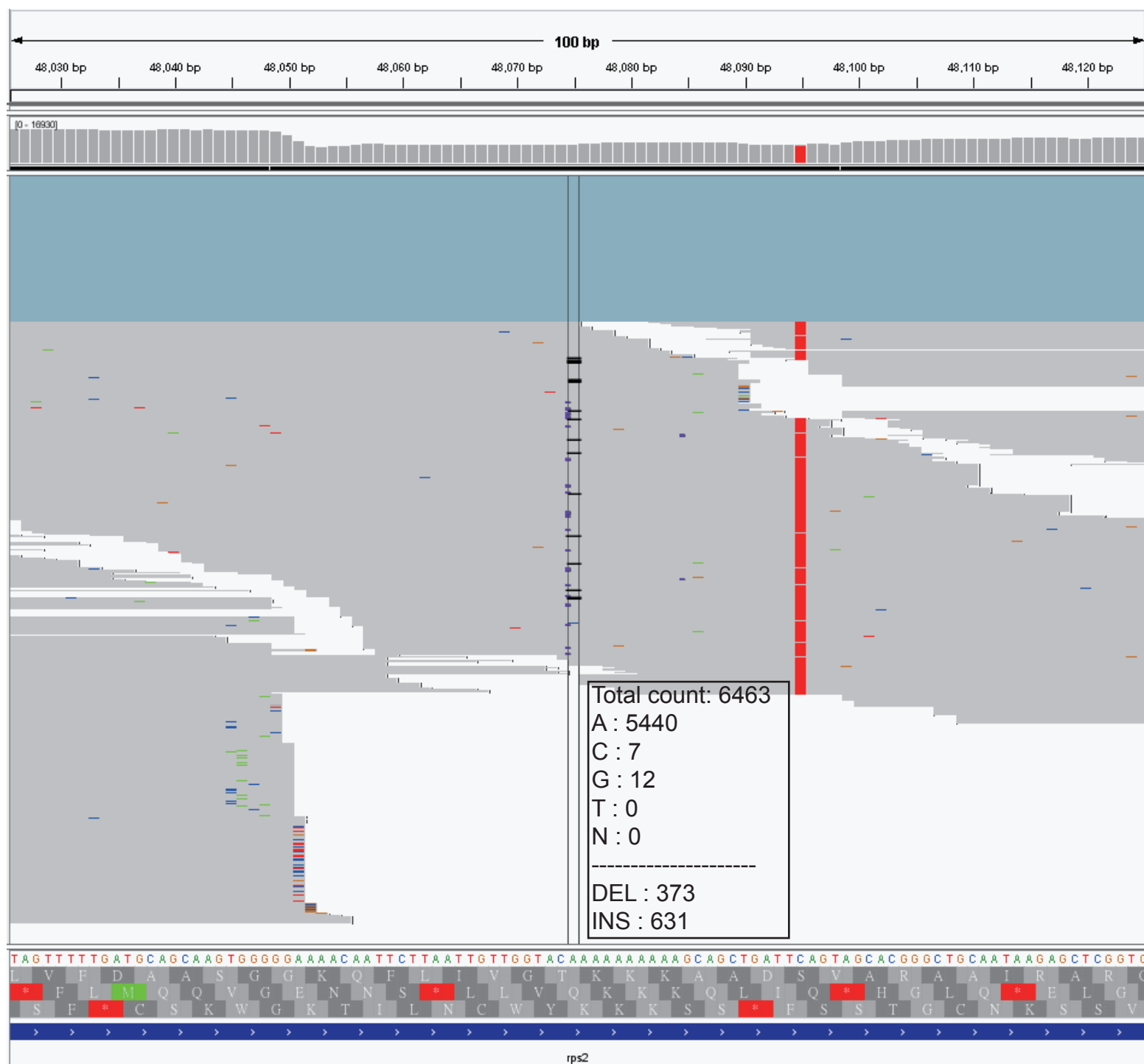

(C) PCR amplicon

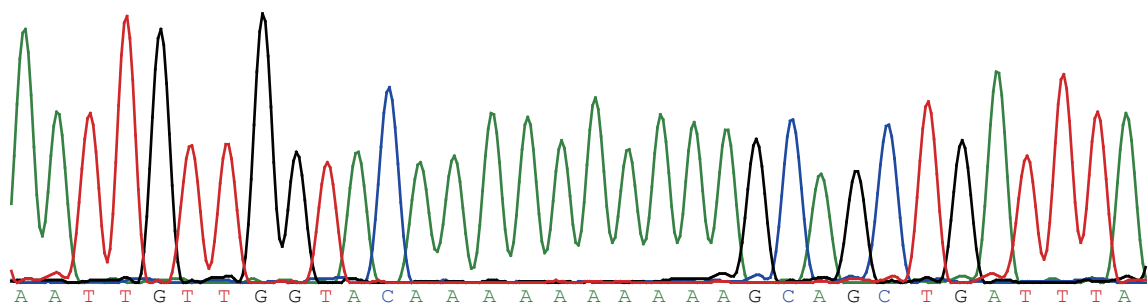

Supplement: S10 Fig — The alignments of WGS (A) and RNA-seq (B) were carried out by bowtie 2 and TopHat2, respectively. PCR amplicon (C) generated from high fidelity Pfu was directly sequenced using ABI PRISM 3730xl. (PDF) [file pone.0129396.s010.pdf]

(A) VR

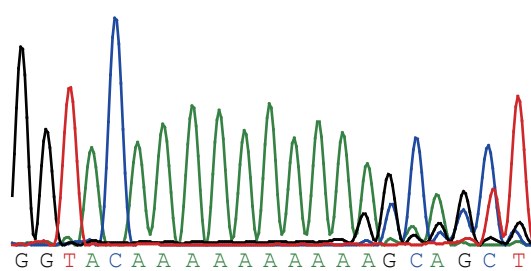

(B) GM

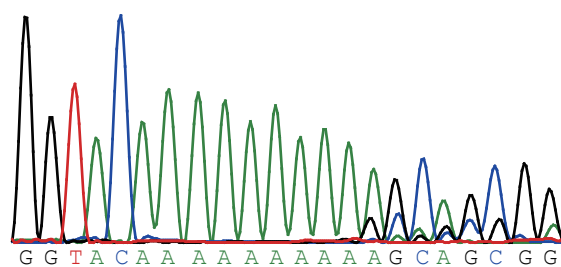

(C) AT

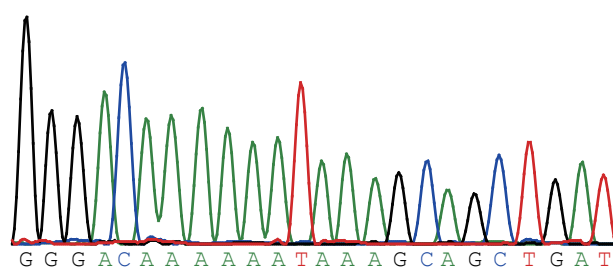

(D) BR

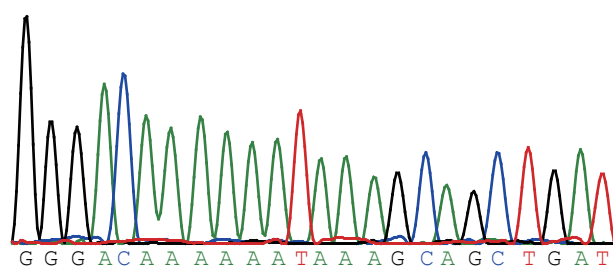

(E) NT

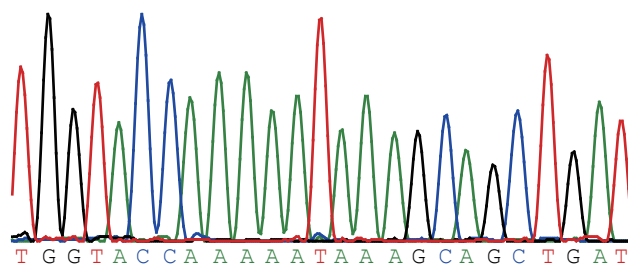

Supplement: S11 Fig — V. radiata (A), G. max (B), A. thaliana (C), B. rapa (D) and N. tabacum (E) were included in the analysis. (PDF) [file pone.0129396.s011.pdf]
