# Supplementary material for: Transcriptional Slippage and RNA Editing Increase the Diversity of Transcripts in Chloroplasts: Insight from Deep Sequencing of Vigna radiata Genome and Transcriptome
Source: PLoS One. 2015 Jun 15;10(6):e0129396. doi: 10.1371/journal.pone.0129396 (PMC4468118; doi:10.1371/journal.pone.0129396)

S2 Fig.

(A) normal transcript without editing

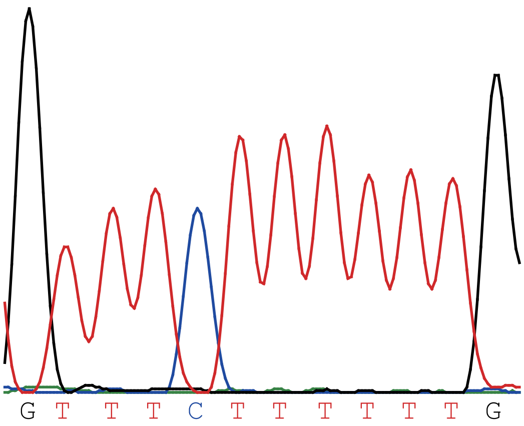


(B) normal transcript with editing

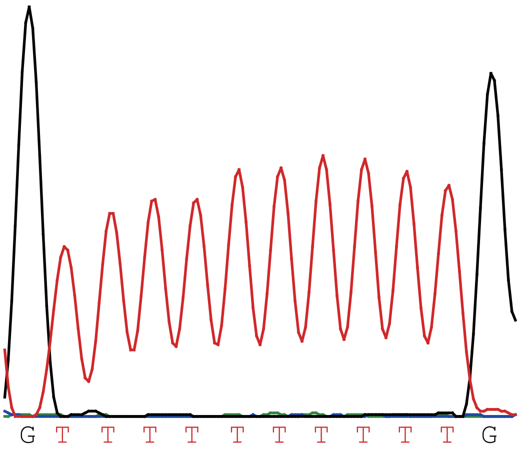


(C) U-deletion transcript without editing

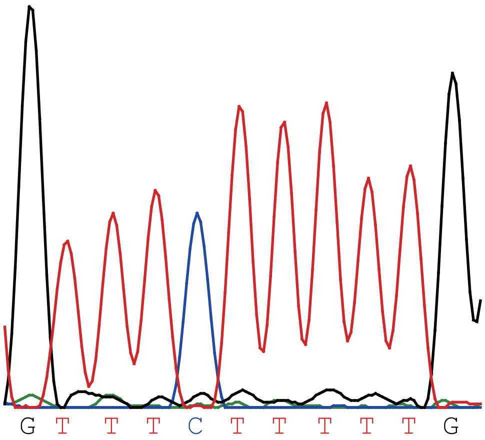


(D) U-delection transcript with editing

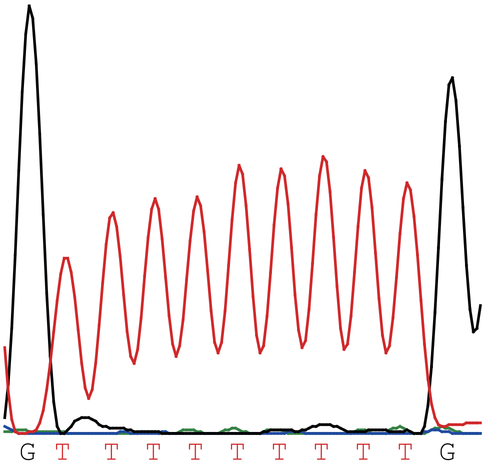


(E) U-insertion transcript with editing

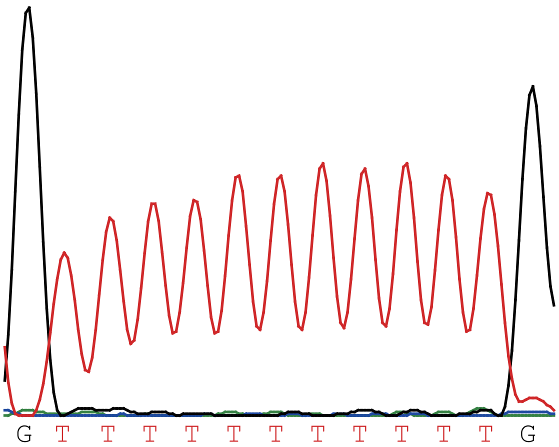

Supplement: S2 Fig — Five kinds of sequences were identified: normal transcripts without (A) or with (B) editing, U-deletion transcripts without (C) or with (D) editing, and U-insertion transcripts with editing (E). (PDF) [file pone.0129396.s002.pdf]
