# Supplementary material for: Transcriptional Slippage and RNA Editing Increase the Diversity of Transcripts in Chloroplasts: Insight from Deep Sequencing of Vigna radiata Genome and Transcriptome
Source: PLoS One. 2015 Jun 15;10(6):e0129396. doi: 10.1371/journal.pone.0129396 (PMC4468118; doi:10.1371/journal.pone.0129396)

S5 Fig.

(A) normal transcript without editing

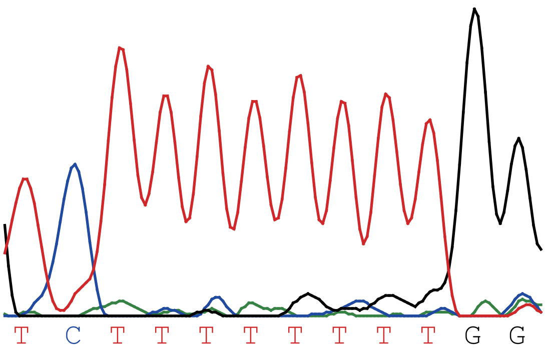


(B) normal transcript with U-to-C editing

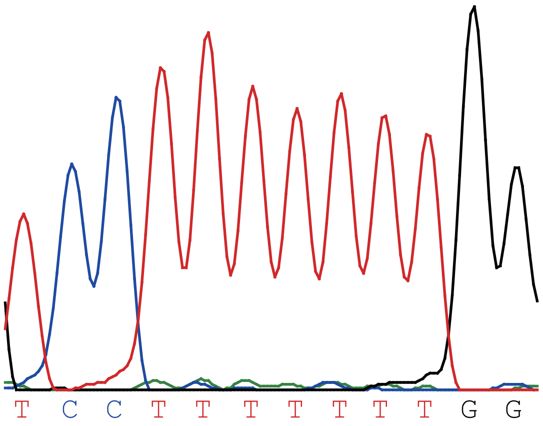


(C) U-deletion transcript without editing

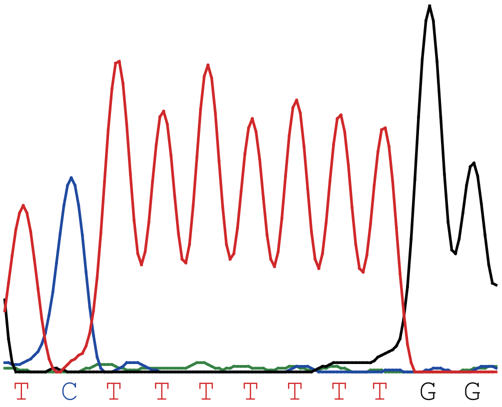


(D) U-insertion transcript without editing

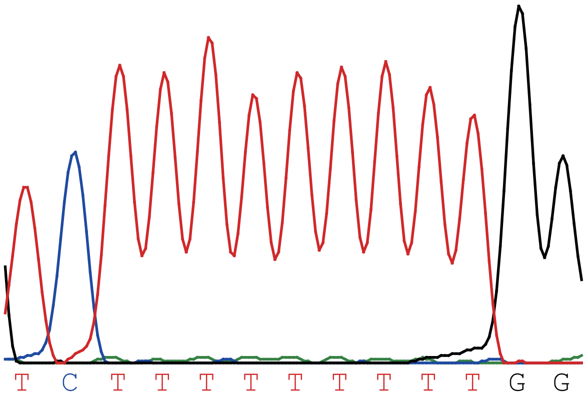

Supplement: S5 Fig — Four kinds of sequences were identified: normal transcripts without (A) or with (B) editing, U-deletion transcripts without (C) editing, and U-insertion transcripts without editing (D). (PDF) [file pone.0129396.s005.pdf]
