# Supplementary material for: Transcriptional Slippage and RNA Editing Increase the Diversity of Transcripts in Chloroplasts: Insight from Deep Sequencing of Vigna radiata Genome and Transcriptome
Source: PLoS One. 2015 Jun 15;10(6):e0129396. doi: 10.1371/journal.pone.0129396 (PMC4468118; doi:10.1371/journal.pone.0129396)
Supplement: S1 Table — (DOC) [file pone.0129396.s012.doc]

### S1 Table. Primers used in this study.

| Species | Primer name | Sequence |
| --- | --- | --- |
| *Vigna radiata, Glycine max*, *Arabidopsis thaliana*, *Brassica rapa*, *Nicotiana tabacum* | m-psbE-F | 5'-gtctggaagcacgggagaacg-3' |
|  | m-psbF-R | 5'-ttgggtttgattgtgtcatagctc-3' |
| *Oryza sativa*, *Zea mays* | r-psbE-F | 5'-gtctggaagcacgggagaacg-3' |
|  | r-psbF-R | 5'-tcgggtttgattgtgtcatagttc-3' |
| *Ginkgo biloba* | g-psbE-F | 5'-gtctgggaatacgggagaacg-3' |
|  | g-psbF-R | 5'-tcgggtttgattgtgtcatagttc-3' |
| *Pinus taiwanesis* | pt-psbE-F | 5'-gtctgggaatacaggagaacg-3' |
|  | pt-psbF-R | 5'-tcgggtttgattgtgtcatatcta-3' |
| *Physcomitrella patens* | pp-psbE-F | 5'-gtctggaaatacaggagagcg-3' |
|  | pp-psbF-R | 5'-ttggatttggttgtgtcatagctt-3' |
| All | d-ndhH-F | 5’-ccbggcggvccwtatgaraatttag-3’ |
|  | d-ndhA-R | 5’-ggrccrgyrtattcaggwccaatacg-3’ |
|  | d-rps2-F | 5’-ttrraagaratgatrsaagcdrgag-3’ |
|  | d-rps2-R | 5’-tcgttaacataccgccgagcc-3’ |
