# Supplementary material for: Transcriptional Slippage and RNA Editing Increase the Diversity of Transcripts in Chloroplasts: Insight from Deep Sequencing of Vigna radiata Genome and Transcriptome
Source: PLoS One. 2015 Jun 15;10(6):e0129396. doi: 10.1371/journal.pone.0129396 (PMC4468118; doi:10.1371/journal.pone.0129396)
Supplement: S2 Table — (DOCX) [file pone.0129396.s013.docx]

**S2 Table.** **Read statistics, error correction and trimming for gDNA samples**

| Sample name | Insert size (bp) | Post error correction and trimming | | CP assembly | | | | |
| --- | --- | --- | --- | --- | --- | --- | --- | --- |
|  |  | No. bases (Gb) | Average read length (bp) | percentage of aligned reads | Coverage | | | |
|  |  |  |  |  | min | max | average | St. Dev. |
| TC1966 | 500 | 3.42 | 100 | 11.44 | 49 | 7938 | 5168.32 | 681.88 |
| NM92 | 500 | 3.80 | 100 | 12.80 | 63 | 7972 | 6409.58 | 795.51 |
| RIL59 | 500 | 15.45 | 100 | 7.58 | 159 | 8047 | 7914.30 | 130.89 |
