# Supplementary material for: Transcriptional Slippage and RNA Editing Increase the Diversity of Transcripts in Chloroplasts: Insight from Deep Sequencing of Vigna radiata Genome and Transcriptome
Source: PLoS One. 2015 Jun 15;10(6):e0129396. doi: 10.1371/journal.pone.0129396 (PMC4468118; doi:10.1371/journal.pone.0129396)
Supplement: S3 Table — (DOC) [file pone.0129396.s014.doc]

S3 Table. Distribution of different repeat types

| Repeat unit | | Repeat length (bp) | | | | | | | | | total |
| --- | --- | --- | --- | --- | --- | --- | --- | --- | --- | --- | --- |
|  |  | 8 | 9 | 10 | 11 | 12 | 13 | 14 | 15 | 16 |  |
| mononucleotide | A/T | 106 | 64 | 17 | 12 | 5 | 1 | 1 | 1 | 1 | 208 |
|  | C/G | 2 | 2 | 0 | 0 | 0 | 0 | 0 | 0 | 0 | 4 |
| dinucleotide | AT/TA | 0 | 0 | 0 | 0 | 1 | 0 | 0 | 0 | 0 | 1 |
