# Supplementary material for: Transcriptional Slippage and RNA Editing Increase the Diversity of Transcripts in Chloroplasts: Insight from Deep Sequencing of Vigna radiata Genome and Transcriptome
Source: PLoS One. 2015 Jun 15;10(6):e0129396. doi: 10.1371/journal.pone.0129396 (PMC4468118; doi:10.1371/journal.pone.0129396)
Supplement: S4 Table — (DOC) [file pone.0129396.s015.doc]

**S4 Table. Summary of single nucleotide variations in *V*. *radiata* var. KPS1 and NM92 with TC1966 used as a reference**.

| Category | Sequence substitution | | TC1966 vs. | |
| --- | --- | --- | --- | --- |
|  | from | to | KPS1 | NM92 |
| transition | A | G | 4 | 3 |
|  | C | T | 5 | 4 |
|  | G | A | 6 | 4 |
|  | T | C | 5 | 2 |
|  | Sum | | 20 | 13 |
| transversion | A | C | 6 | 6 |
|  | A | T | 4 | 3 |
|  | C | A | 18 | 18 |
|  | C | G | 1 | 1 |
|  | G | C | 3 | 3 |
|  | G | T | 14 | 14 |
|  | T | A | 6 | 6 |
|  | T | G | 14 | 14 |
|  | Sum | | 66 | 65 |
