# Supplementary material for: Transcriptional Slippage and RNA Editing Increase the Diversity of Transcripts in Chloroplasts: Insight from Deep Sequencing of Vigna radiata Genome and Transcriptome
Source: PLoS One. 2015 Jun 15;10(6):e0129396. doi: 10.1371/journal.pone.0129396 (PMC4468118; doi:10.1371/journal.pone.0129396)
Supplement: S5 Table — (DOC) [file pone.0129396.s016.doc]

**S5 Table. Summary of indels in *V*. *radiata* var. KPS1 and NM92 with TC1966 used as a reference**.

| Category | Sequence substitution | | TC1966 vs. | |
| --- | --- | --- | --- | --- |
|  | from | to | KPS1 | NM92 |
| indel (1 bp) | - | A | 7 | 2 |
|  | - | T | 7 | 2 |
|  | A | - | 12 | 1 |
|  | T | - | 14 | 3 |
|  | Sum | | 40 | 8 |
| indel (2 bp) | AT | -- | 1 | 1 |
|  | -- | AA | 1 | 0 |
|  | Sum | | 2 | 1 |
