# Supplementary material for: Transcriptional Slippage and RNA Editing Increase the Diversity of Transcripts in Chloroplasts: Insight from Deep Sequencing of Vigna radiata Genome and Transcriptome
Source: PLoS One. 2015 Jun 15;10(6):e0129396. doi: 10.1371/journal.pone.0129396 (PMC4468118; doi:10.1371/journal.pone.0129396)
Supplement: S6 Table — (DOC) [file pone.0129396.s017.doc]

**S6 Table. SSR loci and TS frequencies.**

| SSR number | SSR type | Size | Genome coordinates | | TS reads | Aligned reads | TS frequency | Note |
| --- | --- | --- | --- | --- | --- | --- | --- | --- |
|  |  |  | Start | End |  |  |  |  |
| 1 | (A)8 | 8 | 882 | 889 | 80 | 1681 | 4.76 |  |
| 2 | (A)8 | 8 | 1036 | 1043 | 15 | 533 | 2.81 |  |
| 3 | (A)9 | 9 | 1183 | 1191 | 54 | 568 | 9.51 |  |
| 4 | (T)9 | 9 | 2323 | 2331 | 833 | 5632 | 14.79 | rpl14 |
| 5 | (T)9 | 9 | 3079 | 3087 | 255 | 2762 | 9.23 |  |
| 6 | (A)8 | 8 | 3158 | 3165 | 0 | 1523 | 0.00 |  |
| 7 | (A)8 | 8 | 4901 | 4908 | 595 | 14848 | 4.01 |  |
| 8 | (T)9 | 9 | 5246 | 5254 | 359 | 2267 | 15.84 | matK |
| 9 | (A)8 | 8 | 5788 | 5795 | 67 | 1474 | 4.55 | matK |
| 10 | (A)8 | 8 | 5913 | 5920 | 10 | 226 | 4.42 | matK |
| 11 | (T)8 | 8 | 6281 | 6288 | 185 | 4028 | 4.59 | matK |
| 12 | (A)8 | 8 | 6439 | 6446 | 456 | 4290 | 10.63 | matK |
| 13 | (A)8 | 8 | 6840 | 6847 | 293 | 9681 | 3.03 |  |
| 14 | (T)8 | 8 | 7021 | 7028 | 1177 | 17658 | 6.67 |  |
| 15 | (A)9 | 9 | 7306 | 7314 | 563 | 5505 | 10.23 |  |
| 16 | (T)9 | 9 | 9858 | 9866 | 324 | 3041 | 10.65 |  |
| 17 | (A)8 | 8 | 10024 | 10031 | 164 | 4649 | 3.53 |  |
| 18 | (A)8 | 8 | 10092 | 10099 | 120 | 2477 | 4.84 |  |
| 19 | (A)9 | 9 | 10401 | 10409 | 1406 | 16831 | 8.35 |  |
| 20 | (A)10 | 10 | 10468 | 10477 | 4583 | 31619 | 14.49 | atpB |
| 21 | (A)11 | 11 | 12539 | 12549 | 131 | 355 | 36.90 |  |
| 22 | (T)9 | 9 | 12749 | 12757 | 1115 | 8686 | 12.84 |  |
| 23 | (A)9 | 9 | 13206 | 13214 | 1679 | 19569 | 8.58 |  |
| 24 | (A)9 | 9 | 13264 | 13272 | 1064 | 9613 | 11.07 |  |
| 25 | (A)8 | 8 | 14723 | 14730 | 76 | 2183 | 3.48 |  |
| 26 | (T)8 | 8 | 15551 | 15558 | 0 | 207 | 0.00 |  |
| 27 | (A)9 | 9 | 15566 | 15574 | 0 | 72 | 0.00 |  |
| 28 | (TA)12 | 24 | 15595 | 15618 | 0 | 9 | 0.00 |  |
| 29 | (A)9 | 9 | 15882 | 15890 | 0 | 40 | 0.00 |  |
| 30 | (T)8 | 8 | 16809 | 16816 | 328 | 10365 | 3.16 |  |
| 31 | (T)8 | 8 | 16845 | 16852 | 650 | 9748 | 6.67 |  |
| 32 | (T)15 | 15 | 17129 | 17143 | 26 | 85 | 30.59 |  |
| 33 | (T)8 | 8 | 18633 | 18640 | 166 | 3333 | 4.98 |  |
| 34 | (A)9 | 9 | 19023 | 19031 | 166 | 1899 | 8.74 |  |
| 35 | (T)9 | 9 | 19314 | 19322 | 229 | 1858 | 12.33 |  |
| 36 | (A)9 | 9 | 19335 | 19343 | 348 | 2538 | 13.71 |  |
| 37 | (T)8 | 8 | 19734 | 19741 | 695 | 7439 | 9.34 |  |
| 38 | (A)9 | 9 | 20808 | 20816 | 118 | 1151 | 10.25 |  |
| 39 | (A)8 | 8 | 21527 | 21534 | 247 | 3433 | 7.19 |  |
| 40 | (G)9 | 9 | 22264 | 22272 | 473 | 25363 | 1.86 | psaA |
| 41 | (T)8 | 8 | 26903 | 26910 | 12 | 410 | 2.93 |  |
| 42 | (T)10 | 10 | 27740 | 27749 | 5802 | 26489 | 21.90 |  |
| 43 | (C)9 | 9 | 29517 | 29525 | 510 | 11340 | 4.50 | psbC |
| 44 | (A)8 | 8 | 29552 | 29559 | 675 | 11301 | 5.97 | psbC |
| 45 | (T)8 | 8 | 31399 | 31406 | 1188 | 28324 | 4.19 |  |
| 46 | (T)8 | 8 | 31616 | 31623 | 195 | 5312 | 3.67 |  |
| 47 | (A)8 | 8 | 31840 | 31847 | 77 | 1497 | 5.14 |  |
| 48 | (T)9 | 9 | 31909 | 31917 | 11763 | 81327 | 14.46 |  |
| 49 | (A)8 | 8 | 31998 | 32005 | 3114 | 58353 | 5.34 |  |
| 50 | (T)8 | 8 | 32086 | 32093 | 12 | 437 | 2.75 |  |
| 51 | (A)11 | 11 | 32253 | 32263 | 182 | 632 | 28.80 |  |
| 52 | (A)8 | 8 | 32549 | 32556 | 0 | 41 | 0.00 |  |
| 53 | (T)8 | 8 | 32762 | 32769 | 2 | 430 | 0.47 |  |
| 54 | (T)8 | 8 | 32794 | 32801 | 42 | 671 | 6.26 |  |
| 55 | (T)8 | 8 | 32944 | 32951 | 2 | 655 | 0.31 |  |
| 56 | (T)9 | 9 | 33007 | 33015 | 35 | 386 | 9.07 |  |
| 57 | (T)8 | 8 | 33433 | 33440 | 54 | 889 | 6.07 |  |
| 58 | (A)8 | 8 | 33533 | 33540 | 23 | 495 | 4.65 |  |
| 59 | (T)9 | 9 | 33543 | 33551 | 24 | 527 | 4.55 |  |
| 60 | (T)12 | 12 | 34000 | 34011 | 44 | 188 | 23.40 |  |
| 61 | (T)9 | 9 | 34190 | 34198 | 1546 | 10364 | 14.92 |  |
| 62 | (T)9 | 9 | 34456 | 34464 | 826 | 6541 | 12.63 |  |
| 63 | (T)12 | 12 | 35230 | 35241 | 230 | 518 | 44.40 |  |
| 64 | (T)8 | 8 | 35561 | 35568 | 1 | 123 | 0.81 |  |
| 65 | (A)8 | 8 | 35977 | 35984 | 16 | 563 | 2.84 |  |
| 66 | (A)8 | 8 | 36151 | 36158 | 8 | 774 | 1.03 |  |
| 67 | (T)8 | 8 | 36719 | 36726 | 0 | 70 | 0.00 |  |
| 68 | (A)8 | 8 | 36797 | 36804 | 4 | 91 | 4.40 |  |
| 69 | (A)12 | 12 | 37758 | 37769 | 266 | 1089 | 24.43 | rpoB |
| 70 | (A)8 | 8 | 37866 | 37873 | 175 | 2645 | 6.62 | rpoB |
| 71 | (A)8 | 8 | 40973 | 40980 | 29 | 1157 | 2.51 |  |
| 72 | (A)8 | 8 | 41024 | 41031 | 20 | 421 | 4.75 |  |
| 73 | (T)9 | 9 | 41067 | 41075 | 90 | 799 | 11.26 |  |
| 74 | (T)8 | 8 | 41525 | 41532 | 184 | 2777 | 6.63 |  |
| 75 | (T)8 | 8 | 41721 | 41728 | 267 | 2737 | 9.76 | rpoC1 |
| 76 | (A)9 | 9 | 42926 | 42934 | 64 | 1000 | 6.40 | rpoC1 |
| 77 | (T)8 | 8 | 42940 | 42947 | 68 | 1162 | 5.85 | rpoC1 |
| 78 | (T)8 | 8 | 45450 | 45457 | 94 | 1835 | 5.12 | rpoC2 |
| 79 | (A)11 | 11 | 45591 | 45601 | 1101 | 5326 | 20.67 | rpoC2 |
| 80 | (A)9 | 9 | 45698 | 45706 | 86 | 1555 | 5.53 | rpoC2 |
| 81 | (A)11 | 11 | 45719 | 45729 | 419 | 1501 | 27.91 | rpoC2 |
| 82 | (A)8 | 8 | 45768 | 45775 | 50 | 1607 | 3.11 | rpoC2 |
| 83 | (A)8 | 8 | 46514 | 46521 | 9 | 318 | 2.83 | rpoC2 |
| 84 | (A)9 | 9 | 46648 | 46656 | 292 | 3302 | 8.84 | rpoC2 |
| 85 | (A)8 | 8 | 47516 | 47523 | 236 | 3949 | 5.98 | rpoC2 |
| 86 | (T)8 | 8 | 47693 | 47700 | 415 | 4852 | 8.55 |  |
| 87 | (A)10 | 10 | 48075 | 48084 | 1004 | 6463 | 15.53 | rps2 |
| 88 | (A)8 | 8 | 49696 | 49703 | 160 | 2453 | 6.52 |  |
| 89 | (A)8 | 8 | 49967 | 49974 | 252 | 6925 | 3.64 |  |
| 90 | (T)8 | 8 | 51340 | 51347 | 2833 | 50579 | 5.60 | atpF |
| 91 | (A)8 | 8 | 51703 | 51710 | 217 | 5197 | 4.18 |  |
| 92 | (A)10 | 10 | 54207 | 54216 | 459 | 4662 | 9.85 |  |
| 93 | (A)8 | 8 | 54757 | 54764 | 548 | 14017 | 3.91 |  |
| 94 | (T)8 | 8 | 55628 | 55635 | 30 | 1254 | 2.39 |  |
| 95 | (T)16 | 16 | 55659 | 55674 | 96 | 414 | 23.19 |  |
| 96 | (T)8 | 8 | 55701 | 55708 | 14 | 540 | 2.59 |  |
| 97 | (A)8 | 8 | 55779 | 55786 | 341 | 5834 | 5.85 |  |
| 98 | (A)8 | 8 | 55813 | 55820 | 333 | 8680 | 3.84 |  |
| 99 | (T)8 | 8 | 55846 | 55853 | 563 | 13696 | 4.11 |  |
| 100 | (T)11 | 11 | 55892 | 55902 | 1397 | 9503 | 14.70 |  |
| 101 | (T)10 | 10 | 56011 | 56020 | 2630 | 33844 | 7.77 |  |
| 102 | (T)9 | 9 | 56027 | 56035 | 1730 | 22899 | 7.55 |  |
| 103 | (T)9 | 9 | 56933 | 56941 | 2329 | 30524 | 7.63 |  |
| 104 | (A)9 | 9 | 57106 | 57114 | 0 | 86 | 0.00 |  |
| 105 | (A)13 | 13 | 57486 | 57498 | 8 | 37 | 21.62 |  |
| 106 | (A)9 | 9 | 57639 | 57647 | 104 | 709 | 14.67 |  |
| 107 | (G)8 | 8 | 58337 | 58344 | 9 | 3478 | 0.26 |  |
| 108 | (A)8 | 8 | 58517 | 58524 | 157 | 4147 | 3.79 |  |
| 109 | (T)8 | 8 | 58759 | 58766 | 10 | 179 | 5.59 |  |
| 110 | (T)8 | 8 | 59228 | 59235 | 158 | 2665 | 5.93 | accD |
| 111 | (A)8 | 8 | 60925 | 60932 | 1440 | 28360 | 5.08 | ycf4 |
| 112 | (A)8 | 8 | 61214 | 61221 | 193 | 4677 | 4.13 | ycf4 |
| 113 | (T)9 | 9 | 61474 | 61482 | 2152 | 12885 | 16.70 | ycf4 |
| 114 | (A)9 | 9 | 61868 | 61876 | 387 | 3741 | 10.34 |  |
| 115 | (A)10 | 10 | 62005 | 62014 | 2702 | 15600 | 17.32 | cemA |
| 116 | (A)8 | 8 | 62759 | 62766 | 301 | 6090 | 4.94 |  |
| 117 | (A)9 | 9 | 63290 | 63298 | 928 | 10670 | 8.70 | petA |
| 118 | (A)9 | 9 | 64191 | 64199 | 666 | 7642 | 8.71 |  |
| 119 | (A)8 | 8 | 64249 | 64256 | 409 | 6066 | 6.74 |  |
| 120 | (T)8 | 8 | 65677 | 65684 | 0 | 64 | 0.00 |  |
| 121 | (T)8 | 8 | 66676 | 66683 | 312 | 7076 | 4.41 |  |
| 122 | (A)9 | 9 | 67001 | 67009 | 6917 | 37048 | 18.67 |  |
| 123 | (T)11 | 11 | 67258 | 67268 | 11338 | 19090 | 59.39 |  |
| 124 | (A)8 | 8 | 67440 | 67447 | 265 | 7670 | 3.46 |  |
| 125 | (A)9 | 9 | 67858 | 67866 | 382 | 5177 | 7.38 |  |
| 126 | (A)9 | 9 | 68225 | 68233 | 49 | 631 | 7.77 | rps18 |
| 127 | (A)11 | 11 | 68923 | 68933 | 762 | 3512 | 21.70 |  |
| 128 | (T)8 | 8 | 69395 | 69402 | 105 | 2478 | 4.24 |  |
| 129 | (T)9 | 9 | 70489 | 70497 | 200 | 1486 | 13.46 |  |
| 130 | (T)10 | 10 | 70583 | 70592 | 197 | 1078 | 18.27 |  |
| 131 | (T)11 | 11 | 70666 | 70676 | 236 | 1343 | 17.57 |  |
| 132 | (A)10 | 10 | 70730 | 70739 | 345 | 778 | 44.34 |  |
| 133 | (A)8 | 8 | 70949 | 70956 | 69 | 1406 | 4.91 |  |
| 134 | (A)9 | 9 | 71401 | 71409 | 145 | 1298 | 11.17 |  |
| 135 | (T)9 | 9 | 72149 | 72157 | 161 | 1519 | 10.60 |  |
| 136 | (T)8 | 8 | 74068 | 74075 | 1602 | 38164 | 4.20 |  |
| 137 | (T)8 | 8 | 74205 | 74212 | 1901 | 17893 | 10.62 | psbT |
| 138 | (T)8 | 8 | 75350 | 75357 | 1277 | 29529 | 4.32 |  |
| 139 | (A)9 | 9 | 76479 | 76487 | 389 | 7281 | 5.34 |  |
| 140 | (T)12 | 12 | 76540 | 76551 | 13175 | 17838 | 73.86 |  |
| 141 | (T)10 | 10 | 76982 | 76991 | 9216 | 21575 | 42.72 |  |
| 142 | (T)11 | 11 | 77099 | 77109 | 404 | 907 | 44.54 |  |
| 143 | (A)8 | 8 | 77989 | 77996 | 2 | 126 | 1.59 |  |
| 144 | (T)9 | 9 | 80265 | 80273 | 95 | 1299 | 7.31 |  |
| 145 | (T)9 | 9 | 80387 | 80395 | 127 | 1890 | 6.72 |  |
| 146 | (T)9 | 9 | 81749 | 81757 | 580 | 7666 | 7.57 | rps19 |
| 147 | (A)8 | 8 | 87427 | 87434 | 57 | 1382 | 4.12 | ycf2 |
| 148 | (A)8 | 8 | 89791 | 89798 | 16 | 585 | 2.74 | ycf2 |
| 149 | (A)9 | 9 | 91085 | 91093 | 132 | 1187 | 11.12 |  |
| 150 | (T)10 | 10 | 91278 | 91287 | 375 | 1949 | 19.24 |  |
| 151 | (A)9 | 9 | 91955 | 91963 | 66 | 637 | 10.36 |  |
| 152 | (T)8 | 8 | 96275 | 96282 | 631 | 12848 | 4.91 |  |
| 153 | (T)9 | 9 | 106068 | 106076 | 58 | 1023 | 5.67 |  |
| 154 | (A)8 | 8 | 107207 | 107214 | 377 | 4298 | 8.77 |  |
| 155 | (A)8 | 8 | 107717 | 107724 | 0 | 365 | 0.00 | ndhF |
| 156 | (T)9 | 9 | 108193 | 108201 | 106 | 2418 | 4.38 | ndhF |
| 157 | (A)9 | 9 | 108406 | 108414 | 128 | 1043 | 12.27 | ndhF |
| 158 | (A)8 | 8 | 108927 | 108934 | 111 | 2064 | 5.38 | ndhF |
| 159 | (A)8 | 8 | 109151 | 109158 | 31 | 601 | 5.16 | ndhF |
| 160 | (A)8 | 8 | 109607 | 109614 | 59 | 1100 | 5.36 |  |
| 161 | (A)8 | 8 | 109884 | 109891 | 0 | 234 | 0.00 |  |
| 162 | (A)8 | 8 | 110037 | 110044 | 1457 | 22273 | 6.54 | rpl32 |
| 163 | (A)9 | 9 | 110502 | 110510 | 22 | 262 | 8.40 |  |
| 164 | (A)14 | 14 | 110842 | 110855 | 272 | 850 | 32.00 | ccsA |
| 165 | (T)10 | 10 | 111106 | 111115 | 212 | 838 | 25.30 | ccsA |
| 166 | (A)9 | 9 | 111555 | 111563 | 173 | 2447 | 7.07 |  |
| 167 | (A)8 | 8 | 111597 | 111604 | 46 | 1861 | 2.47 |  |
| 168 | (T)8 | 8 | 111742 | 111749 | 91 | 1918 | 4.74 |  |
| 169 | (T)8 | 8 | 112386 | 112393 | 450 | 10879 | 4.14 | ndhD |
| 170 | (A)9 | 9 | 113375 | 113383 | 4749 | 32579 | 14.58 |  |
| 171 | (T)9 | 9 | 113942 | 113950 | 106 | 689 | 15.38 |  |
| 172 | (T)8 | 8 | 115190 | 115197 | 121 | 2706 | 4.47 |  |
| 173 | (T)8 | 8 | 115232 | 115239 | 27 | 1213 | 2.23 |  |
| 174 | (T)8 | 8 | 115788 | 115795 | 288 | 7691 | 3.74 |  |
| 175 | (T)10 | 10 | 115845 | 115854 | 534 | 4968 | 10.75 |  |
| 176 | (A)11 | 11 | 116593 | 116603 | 451 | 1106 | 40.78 |  |
| 177 | (T)8 | 8 | 117188 | 117195 | 175 | 5347 | 3.27 |  |
| 178 | (A)9 | 9 | 118172 | 118180 | 1007 | 4502 | 22.37 | ndhA |
| 179 | (A)9 | 9 | 119435 | 119443 | 588 | 5050 | 11.64 |  |
| 180 | (T)8 | 8 | 119726 | 119733 | 170 | 4683 | 3.63 |  |
| 181 | (T)8 | 8 | 119878 | 119885 | 0 | 16 | 0.00 |  |
| 182 | (A)10 | 10 | 119945 | 119954 | 0 | 8 | 0.00 |  |
| 183 | (T)9 | 9 | 119999 | 120007 | 0 | 16 | 0.00 |  |
| 184 | (T)9 | 9 | 120268 | 120276 | 168 | 3139 | 5.35 | ycf1 |
| 185 | (T)11 | 11 | 120933 | 120943 | 84 | 555 | 15.14 | ycf1 |
| 186 | (T)11 | 11 | 121419 | 121429 | 78 | 753 | 10.36 | ycf1 |
| 187 | (A)10 | 10 | 121717 | 121726 | 19 | 235 | 8.09 | ycf1 |
| 188 | (T)8 | 8 | 122130 | 122137 | 0 | 112 | 0.00 | ycf1 |
| 189 | (T)8 | 8 | 122241 | 122248 | 2 | 443 | 0.45 | ycf1 |
| 190 | (T)9 | 9 | 122257 | 122265 | 26 | 425 | 6.12 | ycf1 |
| 191 | (T)9 | 9 | 122287 | 122295 | 28 | 555 | 5.05 | ycf1 |
| 192 | (T)12 | 12 | 122612 | 122623 | 40 | 462 | 8.66 | ycf1 |
| 193 | (T)8 | 8 | 122726 | 122733 | 17 | 736 | 2.31 | ycf1 |
| 194 | (T)8 | 8 | 122741 | 122748 | 7 | 766 | 0.91 | ycf1 |
| 195 | (T)10 | 10 | 123107 | 123116 | 189 | 1114 | 16.97 | ycf1 |
| 196 | (T)8 | 8 | 123129 | 123136 | 60 | 1917 | 3.13 | ycf1 |
| 197 | (T)10 | 10 | 123401 | 123410 | 139 | 682 | 20.38 | ycf1 |
| 198 | (T)8 | 8 | 123697 | 123704 | 23 | 550 | 4.18 | ycf1 |
| 199 | (T)8 | 8 | 123944 | 123951 | 0 | 79 | 0.00 | ycf1 |
| 200 | (T)8 | 8 | 124048 | 124055 | 31 | 1053 | 2.94 | ycf1 |
| 201 | (A)9 | 9 | 124327 | 124335 | 13 | 224 | 5.80 | ycf1 |
| 202 | (T)9 | 9 | 124511 | 124519 | 33 | 378 | 8.73 | ycf1 |
| 203 | (G)8 | 8 | 124577 | 124584 | 5 | 306 | 1.63 | ycf1 |
| 204 | (A)8 | 8 | 124753 | 124760 | 7 | 324 | 2.16 | ycf1 |
| 205 | (T)8 | 8 | 124969 | 124976 | 352 | 4630 | 7.60 | ycf1 |
| 206 | (A)9 | 9 | 126107 | 126115 | 58 | 1005 | 5.77 |  |
| 207 | (A)8 | 8 | 135901 | 135908 | 609 | 9830 | 6.20 |  |
| 208 | (T)9 | 9 | 140220 | 140228 | 66 | 604 | 10.93 |  |
| 209 | (A)10 | 10 | 140896 | 140905 | 376 | 1671 | 22.50 |  |
| 210 | (T)9 | 9 | 141090 | 141098 | 132 | 1336 | 9.88 |  |
| 211 | (T)8 | 8 | 142385 | 142392 | 16 | 624 | 2.56 | ycf2 |
| 212 | (T)8 | 8 | 144749 | 144756 | 61 | 1475 | 4.14 | ycf2 |
| 213 | (A)9 | 9 | 150426 | 150434 | 582 | 7567 | 7.69 | rps19 |
| 214^*^ | (A)6 or (A)8 | 6 or 10 | 64839 | 64844 or (64848) | 3846 | 31962 | 12.03 | psbF |

^*^ This locus was not included in the statistical analysis of Fig 2 and the amount of SSRs. The size variance in this locus is due to RE substituting a nucleotide in the position 64845.
