# Supplementary material for: Transcriptional Slippage and RNA Editing Increase the Diversity of Transcripts in Chloroplasts: Insight from Deep Sequencing of Vigna radiata Genome and Transcriptome
Source: PLoS One. 2015 Jun 15;10(6):e0129396. doi: 10.1371/journal.pone.0129396 (PMC4468118; doi:10.1371/journal.pone.0129396)
Supplement: S7 Table — (DOC) [file pone.0129396.s018.doc]

### S7 Table. SRA data used in this study.

| Species | Accession number | Total reads | Alignment rate (%) |
| --- | --- | --- | --- |
| *Vigna radiata* | SRR1867748 | 53046888 | 52.65 |
| *Glycine max* | SRR1371393 | 26435289 | 1.68 |
| *Arabidopsis thaliana* | SRR352212 | 28819618 | 39.48 |
| *Brassica rapa* | SRR935488 | 13855039 | 7.75 |
| *Nicotiana tabacum* | SRR1067863 | 81918191 | 57.01 |
| *Zea mays* | SRR765211 | 310405729 | 0.59 |
| *Oryza sativa* | SRR1449240 | 60883471 | 5.89 |
| *Pinus taeda* | SRR1200435 | 37692638 | 0.42 |
| *Ginkgo biloba* | SRR325161 | 33309579 | 0.84 |
| *Physcomitrella patens* | SRR1589684 | 19767590 | 0.56 |
